# Exacerbating risk in human-ignited large fires over western United States due to lower flammability thresholds and greenhouse gas emissions

**DOI:** 10.1093/pnasnexus/pgaf012

**Published:** 2025-02-11

**Authors:** Fa Li, Qing Zhu, Kunxiaojia Yuan, Huanping Huang, Volker C Radeloff, Min Chen

**Affiliations:** Department of Forest and Wildlife Ecology, University of Wisconsin-Madison, Madison, WI 53706, USA; Climate and Ecosystem Sciences Division, Lawrence Berkeley National Laboratory, Berkeley, CA 94720, USA; Climate and Ecosystem Sciences Division, Lawrence Berkeley National Laboratory, Berkeley, CA 94720, USA; Climate and Ecosystem Sciences Division, Lawrence Berkeley National Laboratory, Berkeley, CA 94720, USA; Department of Geography and Anthropology, Louisiana State University, Baton Rouge, LA 70803, USA; Department of Forest and Wildlife Ecology, University of Wisconsin-Madison, Madison, WI 53706, USA; Department of Forest and Wildlife Ecology, University of Wisconsin-Madison, Madison, WI 53706, USA

**Keywords:** climate change, wildfires, western United States, anthropogenic impacts, human–fire interactions

## Abstract

Large fires in the western United States become highly probable when dry conditions surpass critical thresholds of vapor pressure deficit (VPD_t_). VPD_t_ likely differs between human- and lightning-ignited fires, potentially leading to ignition-type varied responses of fire weather risk to natural variability and various anthropogenic forcings, yet a comprehensive quantification remains lacking. Here, through fire observations with ignition types and a machine learning method, we found that human-ignited large fires had consistently lower thresholds (VPD_t_) across western US ecoregions. Consequently, the annual number of flammable days (when VPD > VPD_t_) for human-caused large fires was 93% higher on average and increased 21% more rapidly than those caused by lightning during 1979–2020. Through robust statistical detection and attribution of Earth System Models, we found that the anthropogenic greenhouse gas (GHG) emissions predominantly (81%) controlled the human-related flammable day increases, which was 18% greater than the effect of GHGs on the increases in lightning-related flammable days. Such ignition-type varied fire weather risk indicates more large fire-prone conditions for human-regulated fire regimes when GHG emissions are enhancing and ignitions are not limited by fuels.

**Significance Statement**

Wildfires in the western United States caused by humans and lightning exhibit distinct characteristics. However, the flammability thresholds (represented here by VPD levels that are sufficiently dry for fire spread) of the two types of fires, and their fire risk responses to natural and anthropogenic forcings, remain unclear. We found that human-ignited large fires showed lower flammability thresholds and 93% more flammable days than those caused by lightning. Flammable days for human-ignited large fires rose 21% faster during 1979–2020. Anthropogenic greenhouse gas emissions contributed 81% to the increases in human-related flammable days, an impact 18% stronger than that for lightning-related flammable day increases. Our findings enhance understanding of how ignition sources and anthropogenic emissions jointly affect western US fire weather risk.

## Introduction

The western United States has experienced marked increases in fire frequency (1), incidence of large fires (2), and fire season length (3, 4) since 2000 compared with the prior two decades. When an ignition occurs and fuels (e.g. living vegetation and dead wood debris) are sufficient and connected, fuel moisture levels play a critical role in determining fire spread (5), extinction (6), and fire sizes (6–8) vapor pressure deficit (VPD), a measure of atmospheric dryness that reflects the difference between actual and saturated air water vapor content, is closely associated with dead and live fuel moisture (7–9), and has emerged as a primary driver behind large fires, such as the catastrophic 2020 fire year in the western United States (4, 6, 10–12). While there is compelling evidence that VPD must surpass a critical threshold (VPD_t_) for sustained combustion (6) and spread of large fires (5), the value of VPD_t_ for large fires, especially across the diverse ecosystems of the western United States, remains elusive. Intriguingly, fires ignited by human activities tend to appear at wetter conditions compared with those ignited by lightning (2, 13). However, the specific VPD_t_ of human- and lightning-ignited large fires, and the degree to which their respective annual numbers of flammable days (we defined a day as “flammable” when its VPD exceeds VPD_t_) have been shaped by climate change due to various anthropogenic and natural forcings (NAT), remain unclear.

Fire regimes in the western United States modulated by humans and lightning exhibit distinct patterns. Lightning-ignited fires mainly occur during the summer when the fuels are adequately dry and typically in mountainous regions with sparse human population (13). In contrast, human-ignited fires are less dependent on fuel aridity and can prolong the fire season almost 3-fold compared with lightning-ignited fires (2, 13–15). These fires are widespread throughout the western United States, especially in areas with moderate development and wildland–urban interfaces (1, 2, 16, 17). The propensity for human-ignited fires even in wetter conditions suggests a fundamental distinction in the prerequisites for fire weather between the two types of fires. Specifically, conditions that are “insufficiently dry” for lightning-ignited fires may still suffice for fires resulting from human activities. This disparity can skew the evaluation of fire weather risk if using a single metric or threshold, as seen in schemes used in the Fire Modeling Intercomparison Project (18) and in the fire danger indices of current early warning systems (19–21). Therefore, pinpointing the specific VPD_t_ for each ignition source is critical for understanding and warning large fires, especially in regions of ecological and economical importance like the western United States, where wildfires are increasingly common.

Furthermore, anthropogenic climate change is intensifying dry conditions, especially when VPD surpasses its critical threshold for large fires. Previous studies have used empirical thresholds, such as using the 90th percentile value of VPD in the study period (4, 22, 23), to quantify the frequency of flammable days with VPD > VPD_t_ (i.e. fire weather risk) and their long-term changes across the western United States (4, 23). These studies found the dominant role of anthropogenic emissions compared with natural variability in fire weather risk increases (4, 23). Anthropogenic emissions include multiple groups of forcing agents such as greenhouse gases (GHGs) and aerosols. Large ensemble simulations from the Community Earth System Model (ESM) reveal that different anthropogenic emissions distinctly influence regional temperature, relative humidity (RH), precipitation, and, consequently, fire weather (24). While GHG emissions that exert warming effects are projected to rise in the future (25), aerosols can either cool or warm the atmosphere and are expected to decline in their emission levels (26, 27). Therefore, disentangling the individual contributions from these anthropogenic and NAT factors is essential for a comprehensive understanding of the changes in fire weather risk and for establishing a scientifically grounded basis to prioritize climate mitigation policies. Yet, this facet remains underexplored. Moreover, the quantification of weather risk, associated with human- and lightning-ignited fires responding to these external forcings, has not been undertaken.

Here, we quantified VPD thresholds for large fires ignited by humans and lightning across ecoregions (28) in the western United States. We defined “large fires” as the largest 10% of fires within each ecoregion, following the methodology of a previous study (2). This percentile-based definition accounts for substantial variations in fire sizes across ecoregions, allowing us to capture a considerable portion of large fires from each ecoregion rather than disproportionately including ecoregions with more frequent large fires (2) (see Materials and methods for more details). We focus on large fires because they represent a substantial share of the total burned area within each ecoregion (2, 13) and have significant impacts on local ecosystems and human communities (2, 29, 30). We used fire data from the Fire Program Analysis fire-occurrence database (31, 32), which explicitly records the causes of fire ignitions. Using a Bayesian inference algorithm, we modeled the relationship between VPD and the probability of large fires and subsequently established the critical VPD threshold (VPD_t_) representing conditions “sufficiently dry” for large fires with a 90% probability (see Materials and methods). Using this VPD_t_, we assessed the frequency and long-term trends of flammable days per year (days when VPD > VPD_t_) for both ignition sources from 1979 to 2020. To delve deeper, we employed the optimal fingerprint algorithm (Materials and methods) and simulations from all accessible ESMs in the Coupled Model Intercomparison Project Phase 6 (CMIP6) (33) and the Detection and Attribution Model Intercomparison Project (DAMIP) (25). We aimed to quantify the individual impacts of GHG emissions, other anthropogenic forcings excluding GHG (OANT), and NAT on observed changes in flammable day frequencies for both human- and lightning-ignited large fires.

## Results

### Lower flammability thresholds and more flammable days for human-ignited large fires

**Fig. 1.**
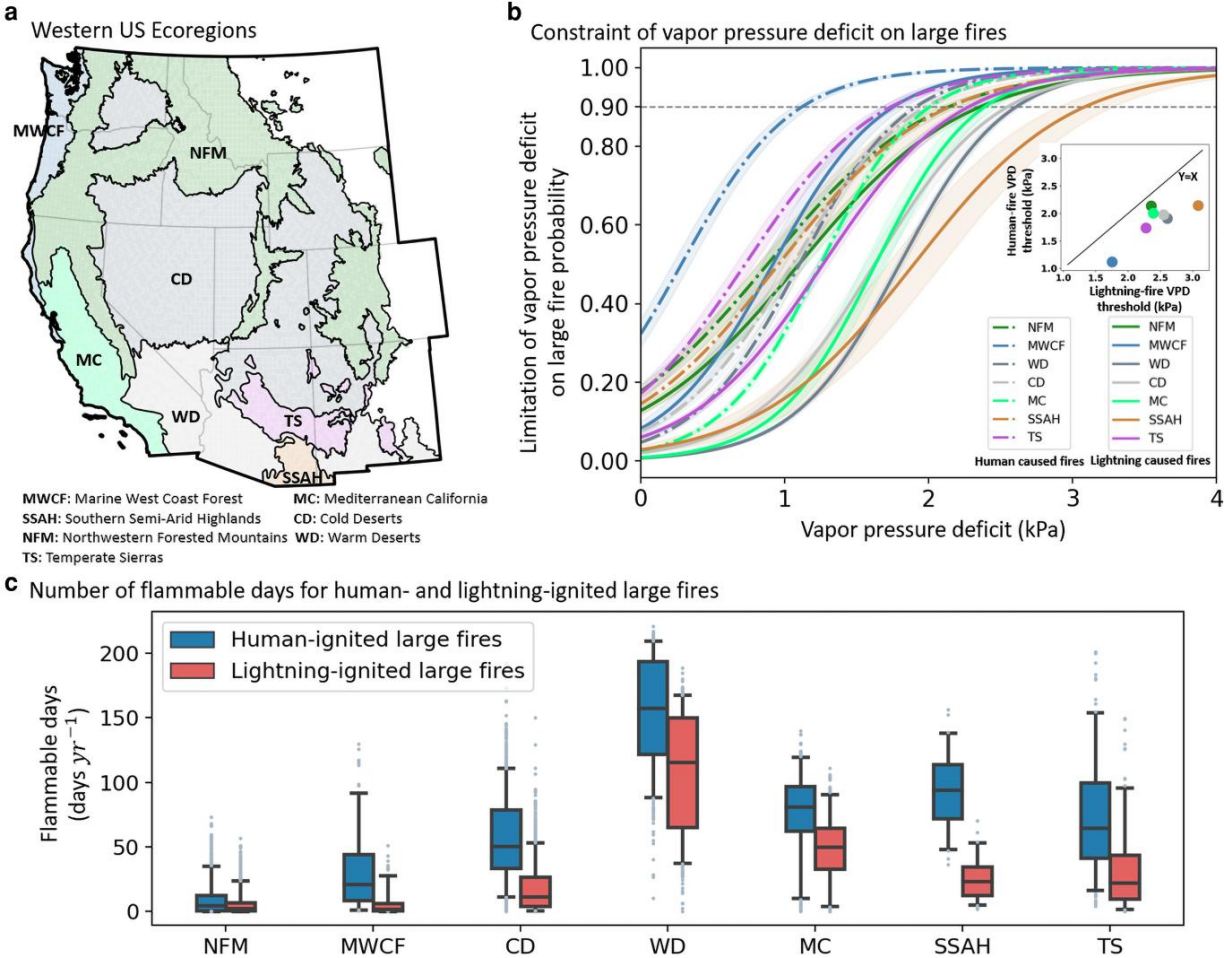
Lower VPD thresholds and more flammable days for human-ignited large fires. The spatial distribution of the ecoregions in the western United States a), the differences in the constraints of VPD on the probability of human- and lightning-ignited large fires in each ecoregion b), and the mean annual number of flammable days from 1979 to 2020 c). In b), lines of the same color represent the response curves of large fire probability to VPD within the same ecoregion for human-ignited (dashed lines) and lightning-ignited fires (solid lines). Shaded ribbons in b) denote the 90% credible intervals for the probability of large fires limited by VPD. The inset within b) displays the VPD thresholds at the 90% probability level of the VPD–large fire logistic function (see Materials and methods). Each point in c) signifies the mean annual number of flammable days (when VPD > VPD_t_) in a grid cell (0.25°). The whiskers in c) represent the 5th and 95th quantiles, with boundaries indicating the 25th, 50th, and 75th quartiles.

We found that human-ignited large fires consistently had a lower VPD_t_ compared with those ignited by lightning across all investigated ecoregions in the western United States (Fig. 1). The estimated VPD_t_ ranged from 1.1 to 2.1 kPa for human-ignited large fires and 1.8 to 3.1 kPa for lightning-ignited large fires (Fig. 1b). Notably, specific ecoregions, including Northwestern Forested Mountains (NFM), Mediterranean California (MC), Warm Deserts (WD), and Southern Semi-Arid Highlands (SSAH) (Fig. 1b), showed higher thresholds (2.0–2.1 kPa) for human-ignited large fires than the other ecoregions. On the contrary, ecoregions such as Cold Deserts (CD), WD, and SSAH exhibited higher VPD thresholds for lightning-ignited fires ranging from 2.6 to 3.1 kPa, considerably surpassing thresholds in other ecoregions, which lay between 1.8 and 2.4 kPa. The persistently lower VPD_t_ (Fig. 1b) implies the greater likelihood for human-ignited large fires to occur even in relatively moist conditions in contrast to lightning-ignited large fires, all else being equal. Given these distinct thresholds, we quantified the annual count of flammable days (days when VPD > VPD_t_) as an indicator of fire weather risk (4, 23, 34) for both human- and lightning-ignited large fires, denoted as *F*_hm_ and *F*_ltn_, respectively.

During 1979–2020, the mean annual *F*_hm_ was about 93% higher than *F*_ltn_ across the western United States. Specifically, around 16% of each year (*F*_hm_ = 58 days year^−1^) presented conditions dry enough for human-ignited large fires, in contrast to a mere 8% (*F*_ltn_ = 30 days year^−1^) for lightning-ignited large fires. A consistent pattern emerged across all ecoregions (Fig. 1c), where *F*_hm_ significantly surpassed *F*_ltn_ (Wilcoxon rank-sum test; *P* < 0.05). The mean *F*_hm_ exceeded *F*_ltn_ by a range of 42 to 500% across different ecoregions (Table S1). Both *F*_hm_ and *F*_ltn_ were typically larger in southern ecoregions (Fig. 1c). For example, in the southern ecoregions such as WD, Temperate Sierras (TS), SSAH, and MC, the mean *F*_hm_ and *F*_ltn_ ranged from 74 to 155 and from 26 to 109 days per year, respectively. This is in contrast to the northern ecoregions such as CD, Marine West Coast Forest-MWCF, and NFM, where *F*_hm_ and *F*_ltn_ ranged from 9 to 57 and from 5 to 18 days annually, respectively (Fig. 1c and Table S1). Our findings underscore the distinct fire weather risk for fires ignited by humans and lightning and highlight the dramatic amplification in the magnitude of flammable days for human-caused large fires.

### More rapid increase of flammable days for human-ignited large fires

**Fig. 2.**
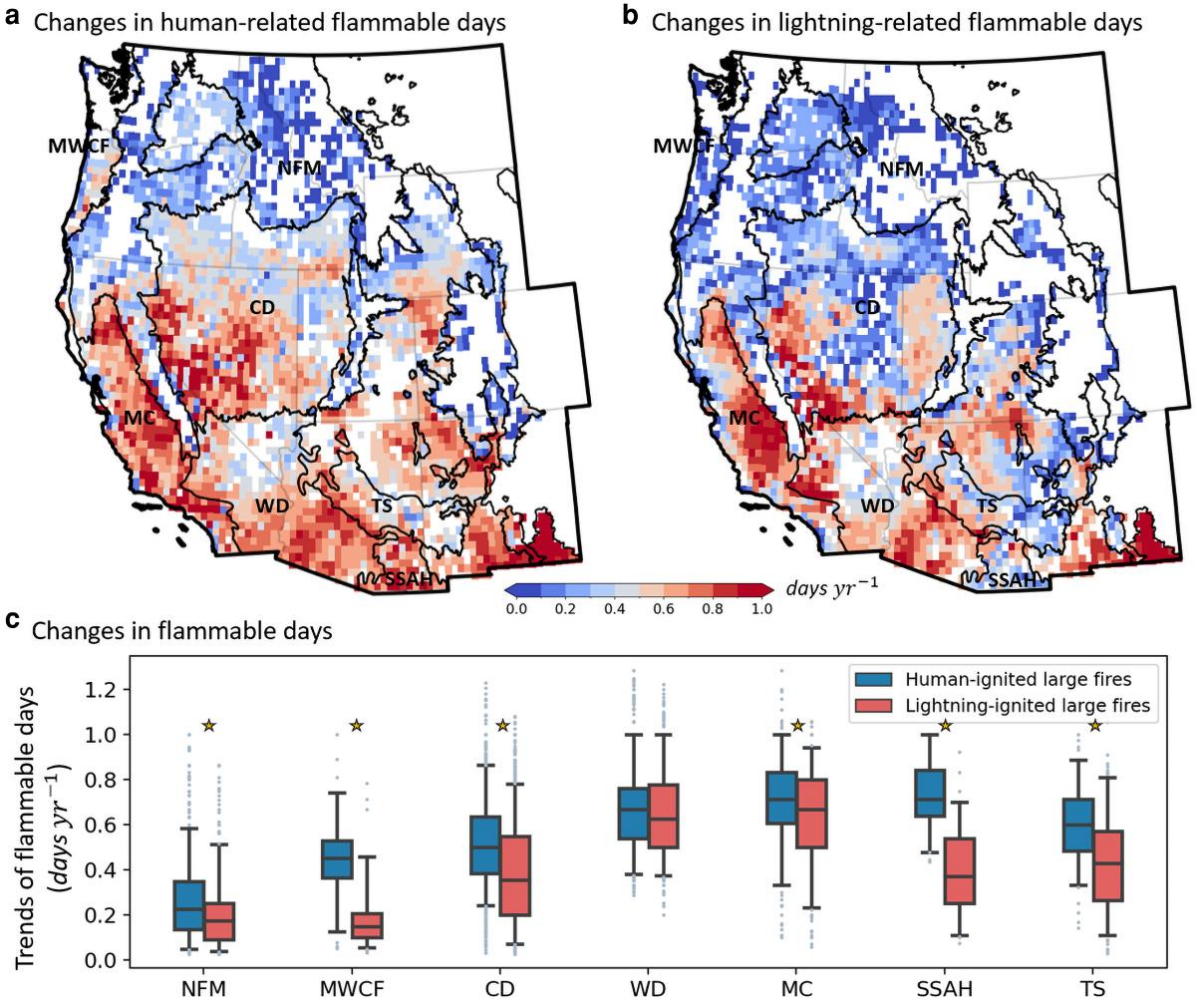
Flammable days above the VPD thresholds for human-ignited large fires increased more rapidly than those for lightning-caused fires in most western US ecoregions from 1979 to 2020. The trends in the frequency of flammable days for human-ignited a) and lightning-ignited large fires b), and their summary statistics for each ecoregion c). Each point in c) signifies the trend of flammable days in a grid cell as shown in a) or b). The whiskers in c) represent 5th and 95th quantiles, with box boundaries representing the 25th, 50th, and 75th quartiles. Gold stars highlight ecoregions where trends of flammable days for human-ignited large fires were significantly (*P* < 0.05) larger than those for lightning-ignited large fires.

We observed evident increases in *F*_hm_ and *F*_ltn_ across the majority of the western United States from 1979 to 2020 (Fig. 2a and b). Specifically, about 66 and 58% of the area in the western United States experienced significant (*P* < 0.05; Materials and methods) increases in *F*_hm_ and *F*_ltn_, respectively. Both *F*_hm_ and *F*_ltn_ trends, influenced by the trends in VPD and VPD_t_, generally decreased with increasing latitudes (Fig. 2a and b). In the southern ecoregions (MC, WD, TS, and SSAH), average *F*_hm_ and *F*_ltn_ trends varied from 0.61 to 0.73 and 0.41 to 0.66 days per year, respectively. These trends were notably higher by 17–170% for *F*_hm_ and 5–230% for *F*_ltn_ compared with the northern ecoregions (NFM, MWCF, and CD). Notably, over 94% of the area in MC located in California experienced an increase in *F*_hm_ and *F*_ltn_ with a trend of 0.71 and 0.64 days year^−1^, translating to 30 and 27 more flammable days since 1979, respectively. The results highlight an amplified urgency to address the rising fire weather risk in the southern ecoregions.

While *F*_hm_ was significantly higher than *F*_ltn_, its rate of increase also outpaced that of *F*_ltn_ across most ecoregions (Fig. 2c). On average, *F*_hm_'s increase across the western United States was 0.52 days year^−1^, about 21% faster than that of *F*_ltn_'s 0.43 days year^−1^. Over the 42-year span, there was an average increase of 22 days for *F*_hm_ across the western United States, ∼4 days more than that for *F*_ltn_. Except for the WD region, *F*_hm_'s increase was significantly > that of *F*_ltn_ in all ecoregions (Wilcoxon’s rank-sum test; *P* < 0.05) (Fig. 2c). The mean trends of *F*_hm_ exceeded *F*_ltn_ by 29 to 125% in the three forest-dominated ecoregions (NFM, MWCF, and TS). In other ecoregions, *F*_hm_ trends were 2–78% larger than *F*_ltn_. These findings indicate that the western United States, irrespective of being forested or not, has seen a more pronounced rise in fire weather risk for human-ignited fires.

**Fig. 3.**
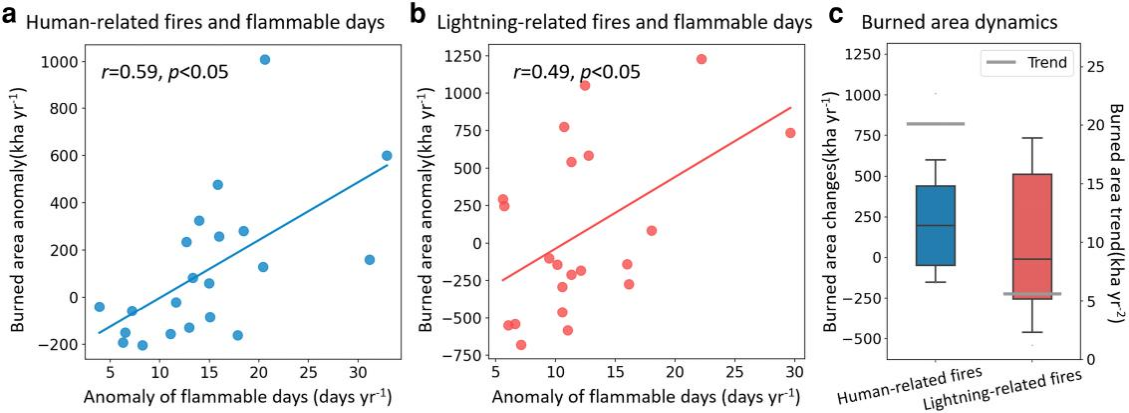
Changes of flammable days are linked to changes in the burned area in the western United States for both ignition types. Significant (*P* < 0.05) relationships between the annual anomaly of burned area and anomaly of annual number of flammable days for human-ignited a) and lightning-ignited large fires during 2000–2020 b). The two-decadal burned area trends (i.e. light gray horizontal lines), and annual burned area changes (i.e. boxplots) from 2011 to 2020 relative to the annual mean burned area during 2000–2010 for human- and lightning-ignited large fires c). The burned area anomaly in a) and b) is calculated by subtracting the mean of burned area during 2000–2011, and solid lines indicate the fitted linear relationships. The whiskers in c) represent the 5th and 95th quantiles, with box boundaries representing the 25th, 50th, and 75th quartiles.

The observed changes in *F*_hm_ and *F*_ltn_ were closely linked to the changes in the burned area across the western United States. Specifically, annual burned area anomaly showed a significantly positive correlation with both *F*_hm_ (*r* = 0.59, *P* < 0.05) and *F*_ltn_ (*r* = 0.49, *P* < 0.05) (Fig. 3a and b). Such positive relationships were also identified in the satellite-observed burned area without differentiating ignition types (Fig. S1). These results suggest a robust association between the fire weather risk and the actual burned area. In conjunction with the increasing flammable days, a positive trend (20.1 kha year^−2^, *P* < 0.1) of burned area was found in human-ignited large fires (Fig. 3c), ∼2.6 times larger than that of lightning-caused large fires (5.6 kha year^−2^, *P* > 0.1). Consequently, the decadal mean annual burned area for human-ignited large fires increased by 242 kha year^−1^, 74% larger than the increase (139 kha year^−1^) of lightning-caused large fires (Fig. 3c). These findings emphasize the pivotal role of the fire weather changes in driving burned area increases in the western United States (10, 23, 35) and highlight the necessity of understanding the rapid changes in fire weather conditions for both ignition types.

### Greater impacts of anthropogenic emissions on rising human-related flammable days

Using ESM simulations from CMIP6 and DAMIP, we investigated the drivers behind the observed increase in flammable days in the western United States (Materials and methods). The multimodel ensemble mean (MMEM) of nine fully coupled and all-forcing-driven ESMs reasonably captured the observed increases in *F*_hm_ and *F*_ltn_. Regionally averaged observations of flammable days generally agreed with that of MMEM (*r* = 0.70 for *F*_hm_ and *r* = 0.68 for *F*_ltn_; *P* < 0.05). The observed trends fell within the 95% CIs (t test) of the ESM-derived trends. From 1979 to 2020, the MMEM showed that average *F*_hm_ increased by 0.50 days year^−1^, about 25% more than *F*_ltn_'s 0.40 days year^−1^. The MMEM of ESM simulations revealed that anthropogenic forcings (GHG and OANT) played a more pronounced role in elevating *F*_hm_ and *F*_ltn_ than NAT (Fig. S2). Specifically, GHG was responsible for 0.26- and 0.24-day annual increases in *F*_hm_ and *F*_ltn_, respectively, whereas OANT accounted for smaller increases of 0.12 and 0.09 days year^−1^ for *F*_hm_ and *F*_ltn_, respectively.

**Fig. 4.**
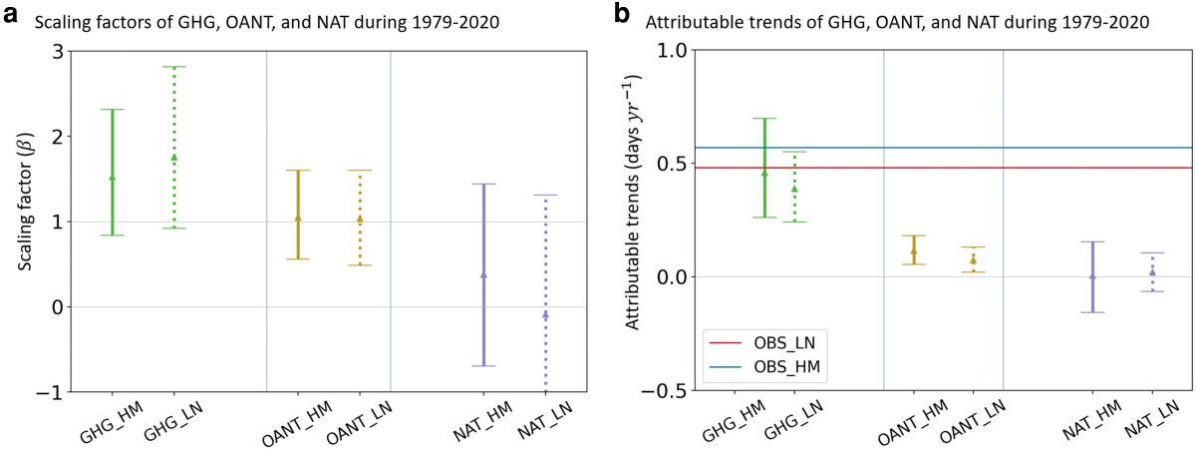
GHG emissions are the dominant drivers of the increasing annual number of flammable days for human- and lightning-ignited large fires over the western United States from 1979 to 2020. Scaling factors derived from regression of observed anomalies in the frequency of flammable days onto three-signal forcings (GHG vs. OANT vs. NAT) for human (HM)- and lighting (LN)-ignited large fires using MMEM a); error bars and triangles show the 5–95% CIs and best estimates of scaling factors; CIs above zero indicate a detectable (*P* < 0.05) forced response, while CIs containing zero indicate an undetectable forced response. Attributable trends for each forcing b); “OBS_LN” and “OBS_HM” represent the observed trends in annual number of flammable days for lightning- and human-ignited large fires, respectively.

Further analysis using a regularized optimal fingerprinting (ROF) algorithm (36, 37) (Materials and methods), which considers climate noise and model structural uncertainties, enabled us to more robustly quantify the relative contributions from GHG, OANT, and NAT to the observed increases in *F*_hm_ and *F*_ltn_. The algorithm regressed observed changes in *F*_hm_ and *F*_ltn_ against model simulations driven by different forcings using scaling factors (Materials and methods). A positive scaling factor with a CI above zero indicates a detectable (*P* < 0.05) forced response. One-signal regressions of observed *F*_hm_ and *F*_ltn_ anomalies against corresponding model simulations driven by all forcings (ALL) revealed detectable fingerprints of MMEM ALL in observations (Fig. S3). Meanwhile, three-signal regressions onto responses driven by GHG, OANT, and NAT highlighted significant (*P* < 0.05) fingerprints of GHG and OANT (Fig. 4a), respectively. For both *F*_hm_ and *F*_ltn_ of MMEM, the 95% CIs of scaling factors for GHG and OANT were above zero (Fig. 4a). The detectability of the GHG and OANT fingerprints was confirmed by most ESMs (Fig. S4a). Compared with GHG and OANT, the ESMs showed greater inconsistency in the detectability of NAT fingerprint with three models indicating insignificant detectability (Fig. S4a). The latter yielded insignificant NAT fingerprints for *F*_hm_ and *F*_ltn_ in the MMEM, as shown by the CIs of its scaling factors including zero (Fig. 4a).

We derived the observed trends of *F*_hm_ and *F*_ltn_ attributable to GHG and OANT by multiplying the scaling factors by the corresponding forced responses simulated by the ESMs (Fig. 4b). The MMEM revealed that GHG contributed to increases of 0.46 (0.26–0.70) days year^−1^ in *F*_hm_ and 0.39 (0.24–0.55) days year^−1^ in *F*_ltn_. All ESMs, except GFDL-ESM4, consistently highlighted the significant positive contributions of GHG to the observed increasing trends of *F*_hm_ and *F*_ltn_. Moreover, a majority of the ESMs (seven of nine) suggested GHG as the primary contributor to the observed trends (Fig. S4b). OANT also made a positive but less discernible contribution to the observed increases, as suggested by the MMEM (Fig. 4b) and seven of the nine ESMs (Fig. S4b). Notably, the impacts of anthropogenic forcings on raising the *F*_hm_ were greater than that of *F*_ltn_ (Fig. 4b). On the contrary, the MMEM indicated no attributable trends of NAT (Fig. 4b). These findings provide a deeper understanding of the distinct responses of fire weather risk to various external forcings for human- vs. lightning-ignited large fires, particularly emphasizing the greater influence of GHGs in shaping fire weather risk changes in the western United States.

## Discussion

Our study unveils two novel and pivotal findings that advance the understanding of fire regimes in the western United States. First, we found that, relative to large fires ignited by lightning, human-caused large fires exhibited lower VPD thresholds with ∼93% more flammable days, and experienced a 21% faster rise in annual number of flammable days. The lower VPD thresholds support previously reported significantly wetter (i.e. higher fuel moisture or lower VPD) occurrence conditions for human-ignited fires than lightning-ignited ones (2, 13). However, this study expands prior investigations by, for the first time, separately quantifying the flammability thresholds for fires of the two ignition types, which are essential for understanding fire dynamics modulated by ignition types and climate (7, 8, 22, 34). Our findings of more frequent flammable days per year for human-ignited large fires are consistent with the prior results showing substantially longer fire seasons for human-ignited fires relative to fires ignited by lightning (1, 13, 38). Founded on a longer temporal period (about four decades) than pioneer studies (1, 2, 13), our results further revealed significant and more rapid increases in the number of flammable days for human-ignited fires than lightning-ignited fires, implying likely faster increase in human-caused large fire frequency than that caused by lightning (1). While the fire weather risk is higher for human-caused large fires and human-caused fires are most detrimental to homes and infrastructures (15, 30), we clarify that the results do not mean lightning-related fire risk is not or less important. Actually, we found that over a half the western US area experienced significant increases in the number of flammable days for lightning-caused fires (Fig. 2) and lightning-ignited fires are generally more intense (1) and linked to nearly two-thirds of fire suppression costs in the United States (30). Instead, our findings highlight the importance of distinguishing fire weather risk based on ignition types.

Second, through robust analyses of multiple ESMs, we found that anthropogenic GHG emissions exerted a dominant influence over other anthropogenic and NAT on the increasing flammable days for both types of large fires between 1979 and 2020. Remarkably, relative to lightning-ignited large fires, the contribution from GHG emissions to the increasing annual number of flammable days for human-ignited large fires was ∼18% larger, indicating higher sensitivity of human-related fire weather risk changes to GHG emissions. Our findings of the major anthropogenic controls on rising fire weather risk confirm the previously reported dominant impact (∼55–88%) from anthropogenic climate change to the increasing fire weather in the western United States through analysis of model simulations and observations (4, 23). Going beyond prior examinations, this study is the first to differentiate the fire weather risk by ignition types, and disentangle the contributions from various external forcings, including GHG emissions, other anthropogenic emissions, and natural variability. Our results therefore establish a more nuanced portrayal of the anthropogenic impacts on dangerous fire weather changes in the western United States.

Overall, our study underscores the critical role of human activities, particularly in the form of GHG emissions and fire ignitions under wetter conditions, on escalating large fire risk in the western US ecoregions. The predominant control from anthropogenic GHG emissions underscores the importance of prioritizing GHG removal and reduction efforts (e.g. through clean energy and natural climate solutions (39–41)) as a primary strategy to mitigate the root causes of long-term increases in fire weather risk. Reducing GHGs is likely to have a more substantial impact on human-regulated fire regimes, given the faster increase in flammable days for human-related fires (Fig. 2c) driven by rising GHGs (Fig. 4b). The lower flammability thresholds of human-caused fires highlight the importance of implementing prevention schemes and regulations for potential human ignitions (e.g. maintenance or setup of powerlines) (42), even under relatively wet weather conditions. Ecological management strategies, encompassing prescribed burning, mechanical fuel treatment, resource allocation, emergency response, and fire suppression (43–46), need to be refined by considering the distinct fire dynamics (e.g. fire frequency and intensity) and flammability (e.g. threshold differences) for human- vs. lightning-ignited large fires (1, 2, 13, 47, 48).

The differences in VPD_t_ for the two types of fires could likely be attributed to the varying fuel conditions during the initial phase of fire expansion and the seasonal differences in ignitions. Similar to the prior study (48), we found significantly higher tree coverage for lightning-caused large fires than for human-caused fires across multiple ecoregions with forests, including NFM, MWCF, MC, and TS (Table S2). With greater tree cover, lightning strikes are more likely to ignite fires from overstory fuels (e.g. live trees) rather than surface fuels (e.g. grasses or dead fine fuels) (49). Overstory fuels tend to be denser and moister than surface live or dead fine fuels (50–52), creating greater impediment during the initial phase of fire spread (48) and requiring drier conditions for sustained fire spread (5, 48). Conversely, human-caused fires are less likely to start from trees and tend to start and spread more easily through surface nonwoody or fine dead wood fuels, even under relatively wetter conditions (2, 48). These initial fuel differences likely lead to the differences in fire flammability thresholds (48).

Furthermore, even with similar initial fuel conditions, lightning-related ignitions mostly occurred in the summer with higher VPD, whereas a remarkable portion of human-caused ignitions can occur in other seasons (e.g. spring and fall) with lower VPD (Fig. S5). The coincidence of higher VPD and lightning ignitions temporally concentrated in summer can lead to higher VPD for lightning-caused large fires and, thus, higher VPD_t_ inferred by the statistical models. While such seasonal differences in fire ignitions may contribute to the observed differences in VPD_t_ (2, 13), the VPD_t_ of human-caused fires should be fundamentally the same as that of lightning-caused fires when they occur in the same season and under the same environmental conditions with the same ignition location (overstory vs. understory). Given the crucial role of VPD in regulating fire dynamics and the limitation of existing data (e.g. relatively coarse ignition location) (31, 53), further exploration of the VPD_t_ differences for the two ignition types is needed when datasets with more precise ignition location (e.g. overstory vs. understory) and detailed fuel characteristics (e.g. fuel size, structure, moisture, type, and chemical composition) (54–57) especially during the initial phase of fire spread (48) are available.

Here, we mainly focused on the controls of VPD on fire weather risk because VPD is a measurement of both environmental dryness and heat, and because of its major link to fire behaviors (e.g. fire spread and extinction) (6, 58, 59), interannual changes of burned area (4, 11, 23), and extreme fire events (10) in the western United States. The significantly lower thresholds of VPD_t_ for human-caused large fires than those related to lightning, and the mean and range of VPD_t_ across ecoregions, were robust, despite the uncertain effects from the proxies of fuel availability and fire suppression (Tables S3 and S4), and different percentile cutoffs for large and small fires (Tables S5 and S6). However, we acknowledge that western US fires are also jointly controlled by other factors, including wind patterns (60, 61), high temperature and droughts (62, 63), heatwaves (64), earlier spring snowmelt (3), winter and spring climate conditions (65), summer precipitation (66), atmospheric humidity (67), and live fuel moisture content (68), topography (69, 70), and forest management (71). A more comprehensive modeling strategy with high physical interpretability (72) that considers all the potential variables, therefore, is needed to understand their instantaneous and time-lagged, direct and indirect, and interactive causal effects on wildfires (73–80). Additionally, this study primarily focused on the impacts of various external forcings on fire weather risk at the interannual scale; deeper understanding of the fire dynamics, therefore, requires further exploration of the anthropogenic climate change impacts on fire ignitions (81–83), fire spread rate (48, 84), and fire extinction (6) across various temporal scales (e.g. daytime vs. nighttime, seasonal vs. interannual scales) (85, 86) and from history to diverse future scenarios.

This study investigated the western United States because wildfires have been rising and are an integral part of the climate mitigation and adaptation solutions there (87, 88). Better understanding of fire mechanisms is essential for more targeted and effective strategies to mitigate the manifold detrimental effects of wildfires on ecosystem functions (e.g. widespread tree mortality (89)), air quality (90), human properties (30) and health (91), and the economy (e.g. ca. $149 billion cost by the 2018 fires in California (92)). While the modeling scheme used here has been applied to understand wildfires globally (6, 93), biased results may be induced when ignoring critical drivers or processes of local wildfires. For example, while in specific areas (e.g. Australia, Amazon forests, and southern Africa), wildfires are greatly controlled by fire season drought (94) or precipitation variations in wet season and teleconnections (e.g. ENSO and Arctic Oscillation) (73, 95–97), fire regimes can also be strongly affected by local fire suppression efforts and fire policies in other regions (e.g. China) (98). Modeling and understanding of wildfires in regions beyond the western United States, therefore, require inclusion of their local major drivers of wildfires rather than simply including VPD. This study gives added importance to understanding the impacts of GHG emissions and ignition types on fire weather risk in the western United States. Since GHG emissions are expected to continue especially under future high-emission scenarios, the interactive impacts of GHG emissions and ignition types on fire risk warrant further exploration in other wildfire hotspots albeit with the spatially heterogeneous nature and complexity of wildfires across different fire regimes globally.

## Materials and methods

### Linking VPD to large fire probability

We used a Bayesian inference approach to assess the relationship between VPD and large fire probability. Ultimately, our objective is to identify the VPD thresholds above which large fires are less likely constrained by VPD after ignitions. Therefore, instead of modeling actual fire occurrence, our models of large fire probability focused on the potential for large fires given an ignition (6, 99–101) by using only data samples where fires already occurred (99–101). Such conditional probability of large fires excludes estimation of the likelihood of ignitions with complexity and stochasticity (99–101), which is similar to widely used fire weather indices (19–21) and has shown reasonable performance in reflecting actual fire activities in the western United States (99, 100). Because fire characteristics (e.g. fire size and frequency) can be ecoregion-varied (Fig. S6) and VPD thresholds can differ among ecosystems (6), we built ecoregion-specific models, each of which was parameterized by a logistic relationship between VPD and large fire probability (6, 93, 102–104) (Eq. 1) where increasing VPD enhances the probability of large fires in a nonlinear manner. Since the climate conditions at the time and place of an ignition are essential for the initial phase of fire expansion (48) and considering the duration of most fires (79%) was one day, we chose the VPD on the start date of a fire at the place of an ignition as the initial climate condition for fire modeling. Additionally, we tested the mean VPD throughout the entire duration of a fire event at the ignition location for fire modeling and found no significant (two-tailed t test, *P* > 0.05) differences in the modeled VPD thresholds. The VPD thresholds were derived using the gridded surface meteorological (gridMET) dataset (105), which has been widely used in analyzing fire dynamics and fire weather changes in the United States (4, 13, 23, 66, 106). When an ignition occurs, in addition to the constraint of VPD (4, 6, 11), fuel insufficiency and human suppression can also prevent the occurrence of large fires. Therefore, we adjusted the fire probabilities by fuel availability and fire suppression (Eq. 2) proxied by net primary productivity (NPP) (1, 22) and population density (PD) (74, 93, 107), respectively. NPP represents the vegetation-accumulated biomass after accounting for ecosystem respiration, and has been commonly used as a proxy of fuel availability (13, 22, 108, 109). Thus, we followed previous studies (13, 22, 108, 109) and used the annual total NPP from MODIS data (110) to represent fuel availability. Additionally, we also tested and discussed the use of aboveground biomass estimated through Global Ecosystem Dynamics Investigation (GEDI) (111) and the annual maximum enhanced vegetation index (EVI) and leaf area index (LAI) from MODIS (112) as fuel availability proxies (99, 113), although current GEDI-based biomass data do not vary across years (111) and the EVI and LAI are generally less used as the fuel availability proxy than NPP in literature as we know (see the Discussion section for more details). Similar to prior studies (74, 93, 107), annual PD was used as the proxy for fire suppression. Besides that, we also tested annual gross domestic product as the fire suppression proxy as utilized in the previous study (114). We first fit the fire models by simultaneously considering the controls of VPD, fuel availability, and fire suppression (Eq. 2). Then, we derived the VPD thresholds based solely on the inferred logistic function between VPD and fires (Eq. 1) since our primary focus is on the limitation of VPD to large fires. This modeling strategy aligns with prior studies aimed at identifying the limitation of VPD on fires or the constraint of fire weather on fires (6, 93, 104, 107). To quantify what is considered “sufficiently dry” where large fires are less likely to be VPD-limited, we chose the 90% posterior probability of the VPD–large fire logistic function as the threshold (VPD_t_) (6).

Specifically, in the *i*th ecoregion (1≤i≤n, where *n* is the total number of ecoregions), the impact of the *j*th control factor $x_{ij}$ (1≤j≤m, where *m* is the count of control factors derived from VPD, NPP, and PD) on large fire probability is modeled as Eq. 1, where $\alpha_{ij}$ is the steepness of the logistic curve and $\beta_{ij}$ is the value at which control factor $x_{ij}$ results in a 50% limitation on fire probability (i.e. $f(x_{ij})=0.5$). We considered $\alpha_{ij}>0$ for VPD and NPP and $\alpha_{ij}<0$ for PD since increasing VPD and fuel availability could increase the probability of large fires while increasing human suppression could reduce that probability (93). For the *i*th ecoregion, the modeled large fire probability, denoted as $F_{i}$, was determined as the product of multiple factors (Eq. 2). This probability links to the observed fire event $Y_{i}$, which is represented through the Bernoulli distribution (Eq. 3). When a large (or small) fire occurred, $Y_{i}$ = equals 1 (or 0), and such binary classification has been commonly represented by Bernoulli distribution (115), achieving reasonable performance in predicting large fire probability (99, 100, 116).


(1) $$f(x_{ij})=\frac{1}{1+e^{-\alpha_{ij}(x_{ij}-\beta_{ij})}}$$

(2) $$F_{i}=\Pi_{\;j=1}^{m}\;f(x_{ij})$$

(3) $$Y_{i}∼\mathrm{Bernoulli}\;(F_{i})$$

### Bayesian model parameter inference

A total of 644,908 fires in the fire database are within the ecoregions studied, and 91.5% of the fire events (589,794) have the ignition causes recorded. To capture a considerable proportion of large fires in each ecoregion, we followed the methodology of a previous study (2) and defined “large fires” as the largest 10% of all fires within a given ecoregion, rather than applying a fixed threshold of fire size across all ecoregions. While both types of fires exhibited a similar skewed distribution with the majority of fires being of small size (Fig. S6), the total number of human-caused fires or large fires can differ considerably by up to a factor of 30 or 21, respectively, compared with lightning-caused fires within the same ecoregion (e.g. MC) (Fig. S7). To ensure data balance for our binary classification (99, 100), we sampled the smallest fires caused by humans (lightning) (Fig. S7), equal to the number of the human-caused (lightning-caused) large fires in each ecoregion as “small fires.” While defining large and small fires is a common step for binary classification of fire sizes, the thresholds used to specify large and small fires can vary across different studies (2, 99, 100, 117–119). A single size threshold is unlikely to reflect the relatively high and low fire risk for all ecoregions (2) since the magnitude of fire sizes varies notably by ecoregions (Fig. S6), likely due to the ecoregion-specific characteristics of fuels, climate, and human effects (2). For example, setting a much higher (lower) threshold for large (small) fires may primarily include ecoregions with a greater number of large (small) fires. In contrast, the ecoregion-percentile-based thresholds consider the magnitude differences across space and the modeled VPD–fire relationship has a clear physical meaning that reflects the fire weather risk transition from high (i.e. using the top 10% largest fires) to low (i.e. using its corresponding smallest fires) controlled by VPD in each ecoregion. To further explore the impacts of percentile cutoffs selected on inferred VPD thresholds, we compared the VPD thresholds derived using the 15 and 20% largest fires and their corresponding smallest fires, to those obtained with the 10% cutoff (see the Discussion section and Tables S5 and S6). Compared with more complicated multiclassification and regression models (73, 74, 120–123), we acknowledge that our binary classification modeling scheme mainly represents a small-to-large fire risk transition that does not explicitly reflect the risk of fires of all sizes.

We used the Bayesian hierarchical modeling framework implemented in the package of PyMC3 (124) to parameterize the models, which allows differences in parameters among ecoregions (i.e. $\alpha_{ij}$ and $\beta_{ij}$) while enabling “partial pooling” that shares the parameter distribution information across different fire events to improve estimates (6, 125). The posterior solutions for the model parameters were inferred using the No-U-Turn Sampler (126), running four chains with 1,000 draws.

### Defining flammable days and frequency of flammable days

For each grid cell, we defined a day as “flammable” if the daily VPD exceeded the VPD threshold (VPD_t_) for the ecoregion. We summed the number of flammable days per year as the frequency of flammable days for human- and lightning-ignited large fires, noted as *F*_hm_ and *F*_ltn_, respectively. For each grid cell, we detected and measured the linear trends in *F*_hm_ and *F*_ltn_ during 1979–2020 using a combination of the Mann–Kendall test and the Theil–Sen slope estimator with temporal autocorrelation in each time series being removed through a variance correction approach (127, 128). We employed the Wilcoxon rank-sum tests (129) to investigate whether significant (*P* < 0.05) differences existed between *F*_hm_ and *F*_ltn_ and between their corresponding trends across grid cells.

### Detection and attribution of trends

We deployed the optimal fingerprint method (37, 130) to detect and attribute the observed trends in *F*_hm_ and *F*_ltn_. The method considers the observed changes (*Y*) to be a linear combination of responses driven by external forcing-driven responses (*X_i_*) and scaling factors ($\beta_{i}$). It also accounts for internal climate noise (ε) within the observations as well as the sampling noise ($\varepsilon_{i}$) that arises from a limited ensemble of model simulations used to derive the forced response (37, 130):


(4) $$Y=\overset{n}{\underset{i=1}{\sum }}\;\beta_{i}(X_{i}-\varepsilon_{i})+\;\varepsilon$$

In our study, *Y* represents the observed interannual anomalies of the number of flammable days, $X_{i}$ refers to the *i*th forcing-driven response, and *n* is the number of forcings (signals). Our forcings included all natural and anthropogenic integrated forcings (ALL), NAT, anthropogenic GHG, and other anthropogenic forcings excluding GHG (OANT = ALL-NAT-GHG). First, we conducted one-signal (*n* = 1) regression to explore whether the signal of ALL was detectable in the observations. Then, we conducted three-signal (*n* = 3) regression to detect and quantify relative contributions from GHG, OANT, and NAT to the observed trends. The internal climate noise (ε) and sampling noise ($\varepsilon_{i}$) were assumed to have the same covariance structure (36) and were estimated with unforced preindustrial model simulations as control (130).

Specifically, we employed the ROF (36) method to estimate the scaling factors in each regression. Different from the standard optimal fingerprint method (37), the regularization that is added to estimate the covariance structure of internal climate variability avoids empirical truncation of orthogonal components for dimension reduction, making it more objective and accurate (36). We selected all ESMs (nine in total, Table S7) in CMIP6 and DAMIP that provided daily RH and surface air temperature, which are required for estimating VPD (Eq. 5) (131, 132). All the model data were regridded to the same spatial resolution (0.25°×0.25°), and we used time series of climate variables during 1979–2020 for attribution analysis. For each time series of anomalies, we derived the 2-year nonoverlapping means to shrink the covariance matrix size by reducing the temporal dimension (130, 133). Subsequently, preindustrial simulations (control experiments) were deployed to estimate the climate noise and two internal variability covariance matrices noted as $C_{1}$ and $C_{2}$ for optimizing the scaling factors and quantifying the uncertainty of the inferred scaling factors (36), respectively.


(5) $$\mathrm{VPD}=0.611\times e^{\frac{17.27T}{\left(T+237.3\right)}}\times \left(1-\frac{\mathrm{RH}}{100}\right)$$

where VPD is the vapor pressure deficit (kPa), *T* is the surface air temperature (^°^C), and RH is the relative humidity.

To better represent the impacts of model uncertainty on the estimate of climate noise, we employed a larger ensemble of preindustrial control simulations (36) (19 ESMs in total, see Table S7). The control simulations for each selected ESM had daily RH and surface air temperature for >420 years. The regionally averaged 420-year time series of flammable days before 1850 from the control simulations of each model were used for trend detection and attribution. For each time series, a 42-year nonoverlapping window was used to divide the entire series into ten subseries. We divided those subseries for each ESM evenly to construct two covariance matrices, $C_{1}$ and $C_{2}$. $C_{1}$ was employed to prewhiten or remove the autocorrelation in the time series of observations (i.e. *Y*) and forced responses (i.e. $X_{i}$) spanning 1979–2020; then, the prewhitened time series of *Y* and $X_{i}$ were used to estimate the optimized scaling factors through the total least square approach (36, 37). $C_{2}$ was utilized to estimate the CI of the scaling factors obtained from the previous step (36, 37). More details on the formulas of the ROF method can be found in the original works in prior publications (36, 37, 134). For a positive scaling factor, if its 90% CI is above 0, then the forced signal is detectable in observations at a 0.05 significant level (*P* < 0.05); if the CI contains 1, the forced response generally agreed well with the observations; if the CI is under (above) 1, the forced response is overestimated (underestimated) and will be scaled down (up) with its scaling factor for trend attribution (37, 130). Negative scaling factors and CI represent opposite signals in model simulations against observations.

## Supplementary Material

pgaf012_Supplementary_Data

## Data Availability

Links to all datasets and model simulations are provided in Tables S8 and S9, respectively. The code is available at https://github.com/GeoAI4GlobalChange/FireWeatherChanges.

## Preprints

This manuscript was posted on a preprint: https://doi.org/10.22541/essoar.172588567.72988193/v3.

## References

[1] Cattau ME , Wessman C, Mahood A, Balch JK. 2020. Anthropogenic and lightning-started fires are becoming larger and more frequent over a longer season length in the USA. Glob Ecol Biogeogr. 29(4):668–681.

[2] Nagy RC , Fusco E, Bradley B, Abatzoglou JT, Balch J. 2018. Human-related ignitions increase the number of large wildfires across US ecoregions. Fire. 1(1):4.

[3] Westerling AL , Hidalgo HG, Cayan DR, Swetnam TW. 2006. Warming and earlier spring increase western US forest wildfire activity. Science. 313(5789):940–943. 16825536 10.1126/science.1128834

[4] Zhuang Y , Fu R, Santer BD, Dickinson RE, Hall A. 2021. Quantifying contributions of natural variability and anthropogenic forcings on increased fire weather risk over the western United States. Proc Natl Acad Sci U S A 118(45):e2111875118. 34725162 10.1073/pnas.2111875118 PMC8609294

[5] Sedano F , Randerson JT. 2014. Multi-scale influence of vapor pressure deficit on fire ignition and spread in boreal forest ecosystems. Biogeosciences. 11(14):3739–3755.

[6] Balch JK , et al 2022. Warming weakens the night-time barrier to global fire. Nature. 602(7897):442–448. 35173342 10.1038/s41586-021-04325-1

[7] Nolan RH , Boer MM, Resco de Dios V, Caccamo G, Bradstock RA. 2016. Large-scale, dynamic transformations in fuel moisture drive wildfire activity across southeastern Australia. Geophys Res Lett. 43(9):4229–4238.

[8] Boer MM , et al 2017. Changing weather extremes call for early warning of potential for catastrophic fire. Earth’s Future. 5(12):1196–1202.

[9] Griebel A , et al 2023. Specific leaf area and vapour pressure deficit control live fuel moisture content. Funct Ecol. 37(3):719–731.

[10] Higuera PE , Abatzoglou JT. 2021. Record-setting climate enabled the extraordinary 2020 fire season in the western United States. Glob Chang Biol. 27(1):1–2. 33048429 10.1111/gcb.15388

[11] Williams AP , et al 2019. Observed impacts of anthropogenic climate change on wildfire in California. Earth's Future. 7(8):892–910.

[12] Seager R , et al 2015. Climatology, variability, and trends in the US vapor pressure deficit, an important fire-related meteorological quantity. J Appl Meteorol Climatol. 54(6):1121–1141.

[13] Balch JK , et al 2017. Human-started wildfires expand the fire niche across the United States. Proc Natl Acad Sci U S A. 114(11):2946–2951. 28242690 10.1073/pnas.1617394114 PMC5358354

[14] Brey SJ , Barnes EA, Pierce JR, Wiedinmyer C, Fischer EV. 2018. Environmental conditions, ignition type, and air quality impacts of wildfires in the southeastern and western United States. Earth's Future. 6(10):1442–1456. 31008140 10.1029/2018EF000972 PMC6472659

[15] Shuman JK , et al 2022. Reimagine fire science for the anthropocene. PNAS Nexus. 1(3):pgac115. 36741468 10.1093/pnasnexus/pgac115 PMC9896919

[16] Syphard AD , et al 2007. Human influence on California fire regimes. Ecol Appl. 17(5):1388–1402. 17708216 10.1890/06-1128.1

[17] Radeloff VC , et al 2005. The wildland–urban interface in the United States. Ecol Appl. 15(3):799–805. 10.1002/eap.2597 35340097

[18] Rabin SS , et al 2017. The fire modeling intercomparison project (FireMIP), phase 1: experimental and analytical protocols with detailed model descriptions. Geosci Model Dev. 10(3):1175–1197.

[19] Jolly WM , Freeborn PH, Page WG, Butler BW. 2019. Severe fire danger index: a forecastable metric to inform firefighter and community wildfire risk management. Fire. 2(3):47.

[20] Van Wagner CE . (1974). *Structure of the Canadian forest fire weather index*. Vol. 1333. Ottawa (ON): Environment Canada, Forestry Service.

[21] Dowdy AJ Mills GA Finkele K de Groot W . (2010). Index sensitivity analysis applied to the Canadian forest fire weather index and the McArthur forest fire danger index. *Meteorol Appl*. 17(3), 298–312.

[22] Ellis TM , Bowman DMJS, Jain P, Flannigan MD, Williamson GJ. 2022. Global increase in wildfire risk due to climate-driven declines in fuel moisture. Glob Chang Biol. 28(4):1544–1559. 34800319 10.1111/gcb.16006

[23] Abatzoglou JT , Williams AP. 2016. Impact of anthropogenic climate change on wildfire across western US forests. Proc Natl Acad Sci U S A. 113(42):11770–11775. 27791053 10.1073/pnas.1607171113 PMC5081637

[24] Touma D , Stevenson S, Lehner F, Coats S. 2021. Human-driven greenhouse gas and aerosol emissions cause distinct regional impacts on extreme fire weather. Nat Commun. 12(1):212. 33431844 10.1038/s41467-020-20570-w PMC7801713

[25] Gillett NP , et al 2016. The detection and attribution model intercomparison project (DAMIP v1. 0) contribution to CMIP6. Geosci Model Dev. 9(10):3685–3697. 10.5194/gmd-9-4521-2016 PMC5911933 29697697

[26] Myhre G , Myhre CL, Samset BH, Storelvmo T. 2013. Aerosols and their relation to global climate and climate sensitivity. Nat Educ Knowl. 4(5):7.

[27] Najafi MR , Zwiers FW, Gillett NP. 2015. Attribution of Arctic temperature change to greenhouse-gas and aerosol influences. Nat Clim Chang. 5(3):246–249.

[28] Omernik JM . 2004. Perspectives on the nature and definition of ecological regions. Environ Manage. 34(S1):S27–S38. 16044553 10.1007/s00267-003-5197-2

[29] Iglesias V , Balch JK, Travis WR. 2022. US fires became larger, more frequent, and more widespread in the 2000s. Sci Adv. 8(11):eabc0020. 35294238 10.1126/sciadv.abc0020 PMC8926334

[30] Mietkiewicz N , et al 2020. In the line of fire: consequences of human-ignited wildfires to homes in the US (1992–2015). Fire. 3(3):50.

[31] Karen C. 2021. *Spatial wildfire occurrence data for the United States 1992-2018 [FPA_FOD_20210617]*. 5th ed. Fort Collins (CO): Forest Service Research Data Archive..

[32] Pourmohamad Y , et al 2024. Physical, social, and biological attributes for improved understanding and prediction of wildfires: FPA FOD-attributes dataset. Earth Syst Sci Data. 16(6):3045–3060.

[33] O'Neill BC , et al 2016. The scenario model intercomparison project (ScenarioMIP) for CMIP6. Geosci Model Dev. 9(9):3461–3482.

[34] Jolly WM , et al 2015. Climate-induced variations in global wildfire danger from 1979 to 2013. Nat Commun. 6:7537. 26172867 10.1038/ncomms8537 PMC4803474

[35] Abatzoglou JT , et al 2021. Projected increases in western US forest fire despite growing fuel constraints. Commun Earth Environ. 2(1):1–8.

[36] Ribes A , Planton S, Terray L. 2013. Application of regularised optimal fingerprinting to attribution. Part I: method, properties and idealised analysis. Clim Dyn. 41(11-12):2817–2836.

[37] Allen MR , Stott PA. 2003. Estimating signal amplitudes in optimal fingerprinting, part I: theory. Clim Dyn. 21(5-6):477–491.

[38] Abatzoglou JT , Balch JK, Bradley BA, Kolden CA. 2018. Human-related ignitions concurrent with high winds promote large wildfires across the USA. Int J Wildland Fire. 27(6):377–386.

[39] Griscom BW , et al 2019. We need both natural and energy solutions to stabilize our climate. Glob Chang Biol. 25(6):1889–1890. 30903637 10.1111/gcb.14612 PMC6646870

[40] Dincer I , Acar C. 2015. A review on clean energy solutions for better sustainability. Int J Energy Res. 39(5):585–606.

[41] Griscom BW , et al 2017. Natural climate solutions. Proc Natl Acad Sci U S A. 114(44):11645–11650. 29078344 10.1073/pnas.1710465114 PMC5676916

[42] Keeley JE , et al 2021. Ignitions explain more than temperature or precipitation in driving Santa Ana wind fires. Sci Adv. 7(30):eabh2262. 34290099 10.1126/sciadv.abh2262 PMC8294765

[43] Kolden CA . 2019. We’re not doing enough prescribed fire in the Western United States to mitigate wildfire risk. Fire. 2(2):30.

[44] Stephens SL , Ruth LW. 2005. Federal forest-fire policy in the United States. Ecol Appl. 15(2):532–542.

[45] Bayham J , Yoder JK. 2020. Resource allocation under fire. Land Econ. 96(1):92–110.

[46] Ager AA , et al 2014. Wildfire exposure and fuel management on western US national forests. J Environ Manage. 145:54–70. 24997402 10.1016/j.jenvman.2014.05.035

[47] Keeley JE , Syphard AD. 2018. Historical patterns of wildfire ignition sources in California ecosystems. Int J Wildland Fire. 27(12):781–799.

[48] Hantson S , Andela N, Goulden ML, Randerson JT. 2022. Human-ignited fires result in more extreme fire behavior and ecosystem impacts. Nat Commun. 13(1):2717. 35581218 10.1038/s41467-022-30030-2 PMC9114381

[49] Outcalt KW . 2008. Lightning, fire and longleaf pine: using natural disturbance to guide management. For Ecol Manage. 255(8-9):3351–3359.

[50] Yebra M , et al 2013. A global review of remote sensing of live fuel moisture content for fire danger assessment: moving towards operational products. Remote Sens Environ. 136:455–468.

[51] Yebra M , Chuvieco E, Riaño D. 2008. Estimation of live fuel moisture content from MODIS images for fire risk assessment. Agric For Meteorol. 148(4):523–536.

[52] Chuvieco E , González I, Verdú F, Aguado I, Yebra M. 2009. Prediction of fire occurrence from live fuel moisture content measurements in a Mediterranean ecosystem. Int J Wildland Fire. 18(4):430–441.

[53] Fusco EJ , et al 2019. Detection rates and biases of fire observations from MODIS and agency reports in the conterminous United States. Remote Sens Environ. 220:30–40.

[54] Gale MG , Cary GJ, Van Dijk AI, Yebra M. 2021. Forest fire fuel through the lens of remote sensing: review of approaches, challenges and future directions in the remote sensing of biotic determinants of fire behaviour. Remote Sens Environ. 255:112282.

[55] Li F , et al 2024. Global impacts of vegetation clumping on regulating land surface heat fluxes. Agric For Meteorol. 345:109820.

[56] Li F , et al 2022. Vegetation clumping modulates global photosynthesis through adjusting canopy light environment. Glob Chang Biol. 29(3):731–746. 36281563 10.1111/gcb.16503 PMC10100496

[57] Zeng Y , et al 2023. Structural complexity biases vegetation greenness measures. Nat Ecol Evol. 7(11):1790–1798. 37710041 10.1038/s41559-023-02187-6

[58] Mueller SE , et al 2020. Climate relationships with increasing wildfire in the southwestern US from 1984 to 2015. For Ecol Manage. 460:117861.

[59] Coop JD , Parks SA, Stevens-Rumann CS, Ritter SM, Hoffman CM. 2022. Extreme fire spread events and area burned under recent and future climate in the western USA. Glob Ecol Biogeogr. 31(10):1949–1959.

[60] Abatzoglou J , et al 2023. Downslope wind-driven fires in the western United States. Earth's Future. 11(5):e2022EF003471.

[61] Mass CF , Ovens D. 2019. The northern California wildfires of 8–9 October 2017: the role of a major downslope wind event. Bull Am Meteorol Soc. 100(2):235–256.

[62] Crockett JL , Westerling AL. 2018. Greater temperature and precipitation extremes intensify western US droughts, wildfire severity, and Sierra Nevada tree mortality. J Clim. 31(1):341–354.

[63] Richardson D , et al 2022. Global increase in wildfire potential from compound fire weather and drought. NPJ Clim Atmos Sci. 5(1):23.

[64] White RH , et al 2023. The unprecedented Pacific northwest heatwave of June 2021. Nat Commun. 14(1):727. 36759624 10.1038/s41467-023-36289-3 PMC9910268

[65] Abolafia-Rosenzweig R , He C, Chen F. 2022. Winter and spring climate explains a large portion of interannual variability and trend in western US summer fire burned area. Environ Res Lett. 17(5):054030.

[66] Holden ZA , et al 2018. Decreasing fire season precipitation increased recent western US forest wildfire activity. Proc Natl Acad Sci U S A. 115(36):E8349–E8357. 30126983 10.1073/pnas.1802316115 PMC6130364

[67] Jain P , Castellanos-Acuna D, Coogan SC, Abatzoglou JT, Flannigan MD. 2022. Observed increases in extreme fire weather driven by atmospheric humidity and temperature. Nat Clim Chang. 12(1):63–70.

[68] Dennison PE , Moritz MA, Taylor RS. 2008. Evaluating predictive models of critical live fuel moisture in the Santa Monica mountains, California. Int J Wildland Fire. 17(1):18–27.

[69] Holsinger L , Parks SA, Miller C. 2016. Weather, fuels, and topography impede wildland fire spread in western US landscapes. For Ecol Manage. 380:59–69.

[70] Alizadeh MR , et al 2021. Warming enabled upslope advance in western US forest fires. Proc Natl Acad Sci U S A 118(22):e2009717118. 34031237 10.1073/pnas.2009717118 PMC8179236

[71] Zald HSJ , Dunn CJ. 2018. Severe fire weather and intensive forest management increase fire severity in a multi-ownership landscape. Ecol Appl. 28(4):1068–1080. 29698575 10.1002/eap.1710

[72] Li F , Chen M, Zhu Q, Yuan K. Physics-guided machine learning of wildfire methane emissions. Artificial Intelligence for Earth System Predictability.

[73] Li F , et al 2023. AttentionFire_v1. 0: interpretable machine learning fire model for burned-area predictions over tropics. Geosci Model Dev. 16(3):869–884.

[74] Zhu Q , et al 2022. Building a machine learning surrogate model for wildfire activities within a global Earth system model. Geosci Model Dev. 15(5):1899–1911.

[75] Kondylatos S , et al 2022. Wildfire danger prediction and understanding with deep learning. Geophys Res Lett. 49(17):e2022GL099368.

[76] Yuan K , et al 2022. Causality guided machine learning model on wetland CH4 emissions across global wetlands. Agric For Meteorol. 324:109115.

[77] Li F , et al 2022. Wetter California projected by CMIP6 models with observational constraints under a high GHG emission scenario. Earth’s Future. 10(4):e2022EF002694.

[78] Yuan K , Zhu Q, Riley WJ, Li F, Wu H. 2022. Understanding and reducing the uncertainties of land surface energy flux partitioning within CMIP6 land models. Agric For Meteorol. 319:108920.

[79] Yuan K , et al 2021. Deforestation reshapes land-surface energy-flux partitioning. Environ Res Lett. 16(2):024014.

[80] Yuan K , et al 2024. Boreal-Arctic wetland methane emissions modulated by warming and vegetation activity. Nat Clim Chang. 14(3):282–288. 38481421 10.1038/s41558-024-01933-3 PMC10927558

[81] Fusco EJ , Abatzoglou JT, Balch JK, Finn JT, Bradley BA. 2016. Quantifying the human influence on fire ignition across the western USA. Ecol Appl. 26(8):2390–2401. 10.1002/eap.1395 27907256

[82] Abatzoglou JT , Kolden CA, Balch JK, Bradley BA. 2016. Controls on interannual variability in lightning-caused fire activity in the western US. Environ Res Lett. 11(4):045005.

[83] Krause A , Kloster S, Wilkenskjeld S, Paeth H. 2014. The sensitivity of global wildfires to simulated past, present, and future lightning frequency. J Geophys Res Biogeosci. 119(3):312–322.

[84] Brown PT , et al 2023. Climate warming increases extreme daily wildfire growth risk in California. Nature. 621(7980):760–766. 37648863 10.1038/s41586-023-06444-3

[85] Turco M , et al 2023. Anthropogenic climate change impacts exacerbate summer forest fires in California. Proc Natl Acad Sci U S A 120(25):e2213815120. 37307438 10.1073/pnas.2213815120 PMC10288651

[86] Luo K , Wang X, de Jong M, Flannigan M. 2023. Drought triggers and sustains overnight fires in North America. Nature. 627(8003):321–327. 10.1038/s41586-024-07028-5 38480963

[87] Fargione JE , et al 2018. Natural climate solutions for the United States. Sci Adv. 4(11):eaat1869. 30443593 10.1126/sciadv.aat1869 PMC6235523

[88] Prichard SJ , et al 2021. Adapting western North American forests to climate change and wildfires: 10 common questions. Ecol Appl. 31(8):e02433. 34339088 10.1002/eap.2433 PMC9285930

[89] Stanke H , Finley AO, Domke GM, Weed AS, MacFarlane DW. 2021. Over half of western United States’ most abundant tree species in decline. Nat Commun. 12(1):451. 33469023 10.1038/s41467-020-20678-z PMC7815881

[90] Carter TS , Heald CL, Selin NE. 2023. Large mitigation potential of smoke PM2. 5 in the US from human-ignited fires. Environ Res Lett. 18(1):014002.

[91] Cascio WE . 2018. Wildland fire smoke and human health. Sci Total Environ. 624:586–595. 29272827 10.1016/j.scitotenv.2017.12.086 PMC6697173

[92] Wang D , et al 2021. Economic footprint of California wildfires in 2018. Nat Sustain. 4(3):252–260.

[93] Kelley DI , et al 2019. How contemporary bioclimatic and human controls change global fire regimes. Nat Clim Chang. 9(9):690–696.

[94] Squire DT , et al 2021. Likelihood of unprecedented drought and fire weather during Australia's 2019 megafires. NPJ Clim Atmos Sci. 4(1):64.

[95] Chen Y , et al 2011. Forecasting fire season severity in South America using sea surface temperature anomalies. Science. 334(6057):787–791. 22076373 10.1126/science.1209472

[96] Andela N , Van Der Werf GR. 2014. Recent trends in African fires driven by cropland expansion and el niño to la niña transition. Nat Clim Chang. 4(9):791–795.

[97] Justino F , Bromwich DH, Schumacher V, daSilva A, Wang SH. 2022. Arctic oscillation and Pacific-North American pattern dominated-modulation of fire danger and wildfire occurrence. NPJ Clim Atmos Sci. 5(1):52.

[98] Hayes JP . 2021. Fire suppression and the wildfire paradox in contemporary China: policies, resilience, and effects in Chinese fire regimes. Hum Ecol. 49(1):19–32.

[99] Gray ME , Zachmann LJ, Dickson BG. 2018. A weekly, continually updated dataset of the probability of large wildfires across western US forests and woodlands. Earth Syst Sci Data. 10(3):1715–1727.

[100] Li J , Albert A, White B, Alok, Mudigonda MA. 2020. Random forest model for the probability of large wildfires in California. International Conference on Learning Representations.

[101] Li F , et al 2024. Projecting large fires in the western US with an interpretable and accurate hybrid machine learning method. Earth's Future. 12(10):e2024EF004588.

[102] Bistinas I , Harrison SP, Prentice IC, Pereira JMC. 2014. Causal relationships versus emergent patterns in the global controls of fire frequency. Biogeosciences. 11(18):5087–5101.

[103] Mann ML , et al 2016. Incorporating anthropogenic influences into fire probability models: effects of human activity and climate change on fire activity in California. PLoS One. 11(4):e0153589. 27124597 10.1371/journal.pone.0153589 PMC4849771

[104] Forkel M , et al 2017. A data-driven approach to identify controls on global fire activity from satellite and climate observations (SOFIA V1). Geosci Model Dev. 10(12):4443–4476.

[105] Abatzoglou JT . 2013. Development of gridded surface meteorological data for ecological applications and modelling. Int J Climatol. 33(1):121–131.

[106] Hawkins LR , Abatzoglou JT, Li S, Rupp DE. 2022. Anthropogenic influence on recent severe autumn fire weather in the west coast of the United States. Geophys Res Lett. 49(4):e2021GL095496.

[107] Li F , Zeng X, Levis S. 2012. A process-based fire parameterization of intermediate complexity in a dynamic global vegetation model. Biogeosciences. 9(7):2761–2780.

[108] Chen B , et al 2021. Climate, fuel, and land use shaped the spatial pattern of wildfire in California's Sierra Nevada. J Geophys Res Biogeosci. 126(2):e2020JG005786.

[109] Hantson S , Lasslop G, Kloster S, Chuvieco E. 2015. Anthropogenic effects on global mean fire size. Int J Wildland Fire. 24(5):589–596.

[110] Zhao M , Heinsch FA, Nemani RR, Running SW. 2005. Improvements of the MODIS terrestrial gross and net primary production global data set. Remote Sens Environ. 95(2):164–176.

[111] Shendryk Y . 2022. Fusing GEDI with earth observation data for large area aboveground biomass mapping. Int J Appl Earth Obs Geoinf. 115:103108.

[112] Shi H , et al 2017. Assessing the ability of MODIS EVI to estimate terrestrial ecosystem gross primary production of multiple land cover types. Ecol Indicators. 72:153–164.

[113] Kahiu MN , Hanan NP. 2018. Fire in sub-saharan Africa: the fuel, cure and connectivity hypothesis. Glob Ecol Biogeogr. 27(8):946–957.

[114] Li F , Levis S, Ward DS. 2013. Quantifying the role of fire in the Earth system—part 1: improved global fire modeling in the Community Earth System Model (CESM1). Biogeosciences. 10(4):2293–2314.

[115] Gyllenberg M , Koski T, Verlaan M. 1997. Classification of binary vectors by stochastic complexity. J Multivar Anal. 63(1):47–72.

[116] Jain P , et al 2020. A review of machine learning applications in wildfire science and management. Environ Rev. 28(4):478–505.

[117] Van Beusekom AE , et al 2018. Fire weather and likelihood: characterizing climate space for fire occurrence and extent in Puerto Rico. Clim Change. 146(1-2):117–131.

[118] Moritz MA . 2003. Spatiotemporal analysis of controls on shrubland fire regimes: age dependency and fire hazard. Ecology. 84(2):351–361.

[119] Gill AM , Allan G. 2008. Large fires, fire effects and the fire-regime concept. Int J Wildland Fire. 17(6):688–695.

[120] Li F , et al 2020. A hierarchical temporal attention-based LSTM encoder-decoder model for individual mobility prediction. Neurocomputing (Amst). 403:153–166. 32501365 10.1016/j.neucom.2020.03.080 PMC7252178

[121] Li F , et al 2018. Big enterprise registration data imputation: supporting spatiotemporal analysis of industries in China. Comput Environ Urban Syst. 70:9–23.

[122] Gui Z , et al 2021. LSI-LSTM: an attention-aware LSTM for real-time driving destination prediction by considering location semantics and location importance of trajectory points. Neurocomputing (Amst). 440:72–88.

[123] Ji F , et al 2024. Unveiling the transferability of PLSR models for leaf trait estimation: lessons from a comprehensive analysis with a novel global dataset. New Phytol. 243(1):111–131. 38708434 10.1111/nph.19807

[124] Salvatier J , Wiecki TV, Fonnesbeck C. 2016. Probabilistic programming in Python using PyMC3. PeerJ Comput Sci. 2:e55. 10.7717/peerj-cs.1516 PMC10495961 37705656

[125] Hobert JP , Casella G. 1996. The effect of improper priors on Gibbs sampling in hierarchical linear mixed models. J Am Stat Assoc. 91(436):1461–1473.

[126] Hoffman MD , Gelman A. 2014. The No-U-turn sampler: adaptively setting path lengths in Hamiltonian Monte Carlo. J Mach Learn Res. 15(1):593–1623.

[127] Sen PK . 1968. Estimates of the regression coefficient based on Kendall's tau. J Am Stat Assoc. 63(324):1379–1389.

[128] Hamed KH , Rao AR. 1998. A modified Mann-Kendall trend test for autocorrelated data. J Hydrol. 204(1-4):182–196.

[129] Cuzick J . 1985. A Wilcoxon-type test for trend. Stat Med. 4(1):87–90. 3992076 10.1002/sim.4780040112

[130] Huang H , Patricola CM, Winter JM, Osterberg EC, Mankin JS. 2021. Rise in northeast US extreme precipitation caused by Atlantic variability and climate change. Weather Clim Extrem. 33:100351.

[131] Abtew W , Melesse A. 2013. Vapor pressure calculation methods. Evap Evapotranspiration Meas Estim. 53–62.

[132] Villalobos FJ , Mateos L, Testi L, Fereres E. 2016. Air temperature and humidity. Princ Agron Sustain Agric. 55–67.

[133] Zhu Z , et al 2016. Greening of the Earth and its drivers. Nat Clim Chang. 6(8):791–795.

[134] Ribes A , Azaïs J-M, Planton S. 2009. Adaptation of the optimal fingerprint method for climate change detection using a well-conditioned covariance matrix estimate. Clim Dyn. 33(5):707–722.

