# Supplementary material for: Exacerbating risk in human-ignited large fires over western United States due to lower flammability thresholds and greenhouse gas emissions
Source: PNAS Nexus. 2025 Feb 11;4(2):pgaf012. doi: 10.1093/pnasnexus/pgaf012 (PMC11812050; doi:10.1093/pnasnexus/pgaf012)
Supplement: pgaf012_Supplementary_Data [file pgaf012_supplementary_data.docx]

*Supplement of*

**Exacerbating risk in human-ignited large fires over western US due to**

**lower flammability thresholds and greenhouse gas emissions**

Fa Li^1,*^, Qing Zhu^2^, Kunxiaojia Yuan^2^, Huanping Huang^3,2^, Volker C. Radeloff^1^, Min Chen^1,*^

^1^ Department of Forest and Wildlife Ecology, University of Wisconsin-Madison, Madison, WI, USA

^2^ Climate and Ecosystem Sciences Division, Lawrence Berkeley National Laboratory, Berkeley, CA, USA

^3^ Department of Geography and Anthropology, Louisiana State University, Baton Rouge, LA, USA

* To whom correspondence may be addressed. Email: fali2@stanford.edu and [min.chen@wisc.edu](mailto:min.chen@wisc.edu)

| **Table S1.** Annual mean number of flammable days for the ecoregions of the western US from 1979 to 2020. | | | |
| --- | --- | --- | --- |
| **Ecoregions** | **Human-related flammable days**  **(mean±std, days yr^-1^)** | **Lightning-related flammable days**  **(mean±std, days yr^-1^)** | **Human increased rate of flammable day (%)** |
| NFM | 9±12 | 6±8 | 50% |
| MWCF | 30±28 | 5±9 | 500% |
| CD | 57±31 | 18±19 | 217% |
| WD | 155±42 | 109±45 | 42% |
| MC | 76±31 | 49±26 | 55% |
| SSAH | 94±29 | 26±16 | 262% |
| TS | 74±43 | 32±31 | 131% |
| Western US | 58±53 | 30±40 | 93% |

| **Table S2.** Mean tree coverage (%) for human- and lightning-ignited large fires, and statistical difference using one-tailed *t*-test. | | | |
| --- | --- | --- | --- |
| Ecoregions | Human-related large fires | Lightning-related large fires | *p-*value |
| NFM** | 31.3 | 33.4 | *P*<0.01 |
| MWCF* | 52.4 | 57.1 | *P*<0.05 |
| CD | 12.1 | 10.5 | *P>*0.1 |
| WD | 8.4 | 5.7 | *P>*0.1 |
| MC** | 15.3 | 22.0 | *P*<0.01 |
| SSAH | 9.9 | 9.2 | *P>*0.1 |
| TS** | 6.2 | 9.3 | *P*<0.01 |

Ecoregions denoted by ** and * signify significantly greater tree coverage in lightning-ignited large fires than those caused by humans with *P*<0.01 and *P*<0.05, respectively.

| **Table S3.** VPD thresholds (kPa) for human- and lightning-ignited large fires using different proxies of fuel availability and fire suppression. | | | |
| --- | --- | --- | --- |
| Proxies | Thresholds for human-related fires  (mean$\pm$std) | Thresholds for lightning-related fires  (mean$\pm$std) | *p-*value |
| NPP+Popu | 1.9$\pm0.3$ | 2.4$\pm$0.4 | *P*<0.05 |
| NPP+GDP | 1.9$\pm0.3$ | 2.5$\pm$0.5 | *P*<0.05 |
| Biomass+Popu | 1.9$\pm0.3$ | 2.4$\pm$0.4 | *P*<0.05 |
| EVI+Popu | 1.7$\pm0.2$ | 2.4$\pm$0.3 | *P*<0.05 |
| LAI+Popu | 1.8$\pm0.3$ | 2.4$\pm$0.4 | *P*<0.05 |

Here *P*<0.05 represents that the VPD thresholds for lightning-ignited large fires are significantly larger than those for human-caused large fires with a one-tailed *t*-test.

| **Table S4.** Statistical comparison of VPD thresholds using different proxies of fuel availability and fire suppression. | | | | |
| --- | --- | --- | --- | --- |
| Proxies | VPD thresholds (kPa) | | | Difference compared with proxies of  NPP+Popu (two-tailed *t*-test, *p*-value) |
|  | mean | minimum | maximum |  |
| NPP+Popu | 2.1 | 1.1 | 3.1 | *---* |
| NPP+GDP | 2.2 | 1.3 | 3.1 | *P>*0.1 |
| Biomass+Popu | 2.1 | 1.2 | 2.8 | *P>*0.1 |
| EVI+Popu | 2.0 | 1.3 | 2.8 | *P>*0.1 |
| LAI+Popu | 2.1 | 1.2 | 2.7 | *P>*0.1 |

| **Table S5.** VPD thresholds (kPa) for human- and lightning-ignited large fires using different percentile cutoffs for large and small fires. | | | |
| --- | --- | --- | --- |
| Percentile cutoffs | Thresholds for human-related fires  (mean$\pm$std) | Thresholds for lightning-related fires  (mean$\pm$std) | *p-*value |
| 10% | 1.9$\pm0.3$ | 2.4$\pm$0.4 | *P*<0.05 |
| 15% | 1.9$\pm0.3$ | 2.4$\pm$0.4 | *P*<0.05 |
| 20% | 1.9$\pm0.4$ | 2.4$\pm$0.5 | *P*<0.05 |

Here *P*<0.05 represents that the VPD thresholds for lightning-ignited large fires are significantly larger than those for human-caused large fires with one-tailed *t*-test.

| **Table S6.** Statistical comparison of VPD thresholds using different percentile cutoffs for large and small fires. | | | | |
| --- | --- | --- | --- | --- |
| Percentile cutoffs | VPD thresholds (kPa) | | | Difference compared with of the 10% percentile cutoff (two-tailed *t*-test, *p*-value) |
|  | mean | minimum | maximum |  |
| 10% | 2.1 | 1.1 | 3.1 | *---* |
| 15% | 2.2 | 1.4 | 2.9 | *P>*0.1 |
| 20% | 2.1 | 1.3 | 2.8 | *P>*0.1 |

| **Table S7:** Earth system models in CMIP6 and DAMIP used for trend attribution. | |
| --- | --- |
| **Model Name** | **Simulations** |
| ACCESS-CM2 (Bi et al., 2020) | historical, SSP585, hist-nat,hist-GHG, piControl (hurs, tas) |
| ACCESS-ESM1-5 (Ziehn et al., 2020) | historical, SSP585, hist-nat, hist-GHG, piControl (hurs, tas) |
| CanESM5 (Swart et al., 2019) | historical, SSP585, hist-nat, hist-GHG, piControl (hurs, tas) |
| CESM2-WACCM (Danabasoglu et al., 2020) | piControl (hurs, tas) |
| CESM2 (Danabasoglu et al., 2020) | piControl (hurs, tas) |
| FGOALS-g3 (Li et al., 2020) | historical, SSP585, hist-nat, hist-GHG (hurs, tas) |
| MIROC6 (Tatebe et al., 2019) | historical, SSP585, hist-nat, hist-GHG, piControl (hurs, tas) |
| MRI-ESM2-0 (Yukimoto et al., 2019) | historical, SSP585, hist-nat, hist-GHG (hurs, tas) |
| NorESM2-LM (Seland et al., 2020) | historical, SSP585, hist-nat, hist-GHG, piControl (hurs, tas) |
| NorESM2-MM (Seland et al., 2020) | piControl (hurs, tas) |
| GFDL-CM4 (Held et al., 2019) | piControl (hurs, tas) |
| EC-Earth3 (Döscher et al., 2021) | piControl (hurs, tas) |
| INM-CM5-0 (Volodin et al., 2019) | piControl (hurs, tas) |
| INM-CM4-8 (Volodin et al., 2019) | piControl (hurs, tas) |
| IPSL-CM6A-LR (Boucher et al., 2020) | historical, SSP585, hist-nat, hist-GHG, piControl (hurs, tas) |
| GFDL-ESM4 (Held et al., 2019) | historical, SSP585, hist-nat, hist-GHG, piControl (hurs, tas) |
| CMCC-CM2-SR5 (Cherchi et al., 2019) | piControl (hurs, tas) |
| CMCC-ESM2 (Lovato et al., 2022) | piControl (hurs, tas) |
| MPI-ESM1-2-HR (Mauritsen et al., 2019) | piControl (hurs, tas) |
| MPI-ESM1-2-LR (Mauritsen et al., 2019) | piControl (hurs, tas) |
| MPI-ESM-1-2-HAM (Mauritsen et al., 2019) | piControl (hurs, tas) |

| **Table S8.** Datasets used in this study. | | |
| --- | --- | --- |
| **Datasets** | **Variables** | **Data Access** |
| gridMET | Vapor Pressure Deficit (VPD) | http://www.climatologylab.org/gridmet.html |
| Fire Program Analysis fire occurrence database | Burned area, ignition date, ignition location (latitude, longitude), ignition source (human or lightning) | <https://www.fs.usda.gov/rds/archive/Catalog/RDS-2013-0009.5> https://www.fs.usda.gov/rds/archive/catalog/RDS-2013-0009.6 |
| Monitoring Trends in Burn Severity (MTBS) | Burned area, burned date | https://www.mtbs.gov/direct-download |
| MODIS MYD17A3HGF V6.1 product | Net Primary Productivity | https://doi.org/10.5067/MODIS/MYD17A3HGF.061 |
| Gridded Population of the World Version 4 (GPWv4) | Population Density | https://sedac.ciesin.columbia.edu/data/collection/gpw-v4 |
| Gridded global datasets for Gross Domestic Product | Gross Domestic Product | https://zenodo.org/records/4972425 |
| Global Ecosystem Dynamics Investigation (GEDI) L4B product | Aboveground Biomass | https://daac.ornl.gov/GEDI/guides/GEDI_L4B_Gridded_Biomass_V2_1.html |
| MODIS MCD43A4 product | Enhanced Vegetation Index | https://developers.google.com/earth-engine/datasets/catalog/MODIS_MCD43A4_006_EVI |
| MODIS MOD15A2H V6.1 product | Leaf Area Index | https://developers.google.com/earth-engine/datasets/catalog/MODIS_061_MOD15A2H |
| MODIS Vegetation Continuous Fields (VCF) product | Tree coverage percent | https://developers.google.com/earth-engine/datasets/catalog/MODIS_006_MOD44B |

| **Table S9.** Model simulations used in this study. | |
| --- | --- |
| **Datasets** | **Data Access** |
| CMIP6 | https://esgf-node.llnl.gov/projects/cmip6/ |
| DAMIP | https://esgf-node.llnl.gov/projects/cmip6/ |


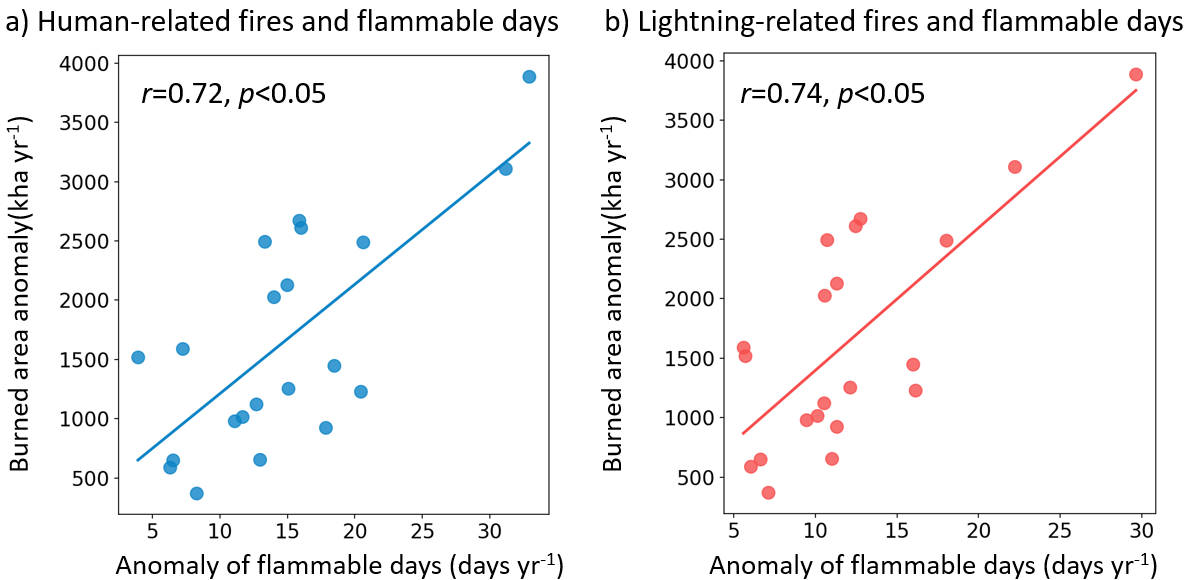


**Fig. S1**| **Changes of flammable days are linked to changes in the total burned area in the western US.** Significant (*p*<0.05) relationships between the annual anomaly of burned area and anomaly of annual number of flammable days for human-ignited **(a)** and lightning-ignited large fires **(b)**. The burned area anomaly in (a) and (b) is calculated by subtracting the mean of burned area during 2000-2011, and solid lines indicate the fitted linear relationships. Here satellite-observed burned area data, Monitoring Trends in Burn Severity (MTBS), is used.


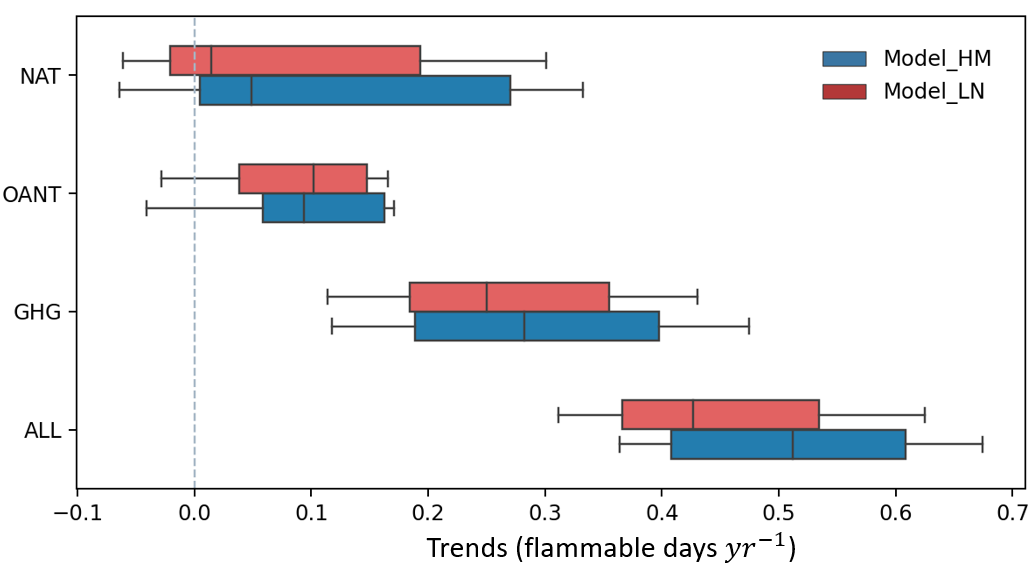


**Fig. S2**| Trends of the annual number of flammable days for human- and lightning-ignited large fires driven by all forcings (ALL), greenhouse gas emissions (GHG), other anthropogenic forcings excluding GHG (OANT), and natural forcings (NAT) over the western US from 1979 to 2020. ‘Model_LN’ and ‘Model_HM’ represent modeled trends in annual flammable days for lightning- and human-ignited large fires, respectively; whiskers represent 5^th^ and 95^th^ quantiles, and boundaries of boxes represent 25^th^, 50^th^, and 75^th^ quartiles.


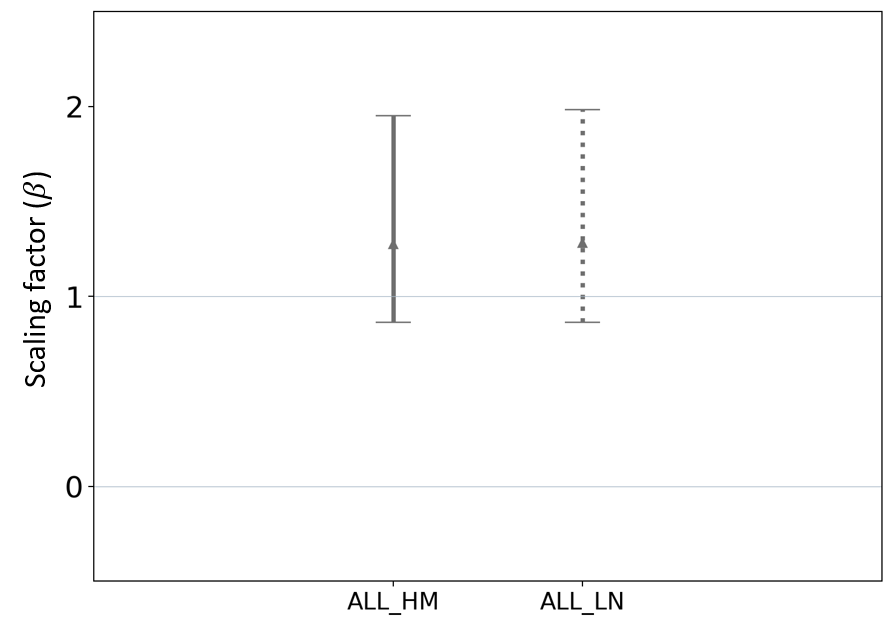


**Fig. S3**| Scaling factors derived from regression of observed anomalies in the frequency of flammable days onto model simulations driven by all forcings for multi-model ensemble mean. Error bars and triangles show the 5%-95% confidence interval and best estimates of scaling factors. Confidence intervals above zero indicate a detectable (*p*<0.05) forced response, while confidence intervals containing zero indicate an undetectable forced response. ‘LN’ and ‘HM’ represent scaling factors for lightning- and human-ignited large fires, respectively.

**
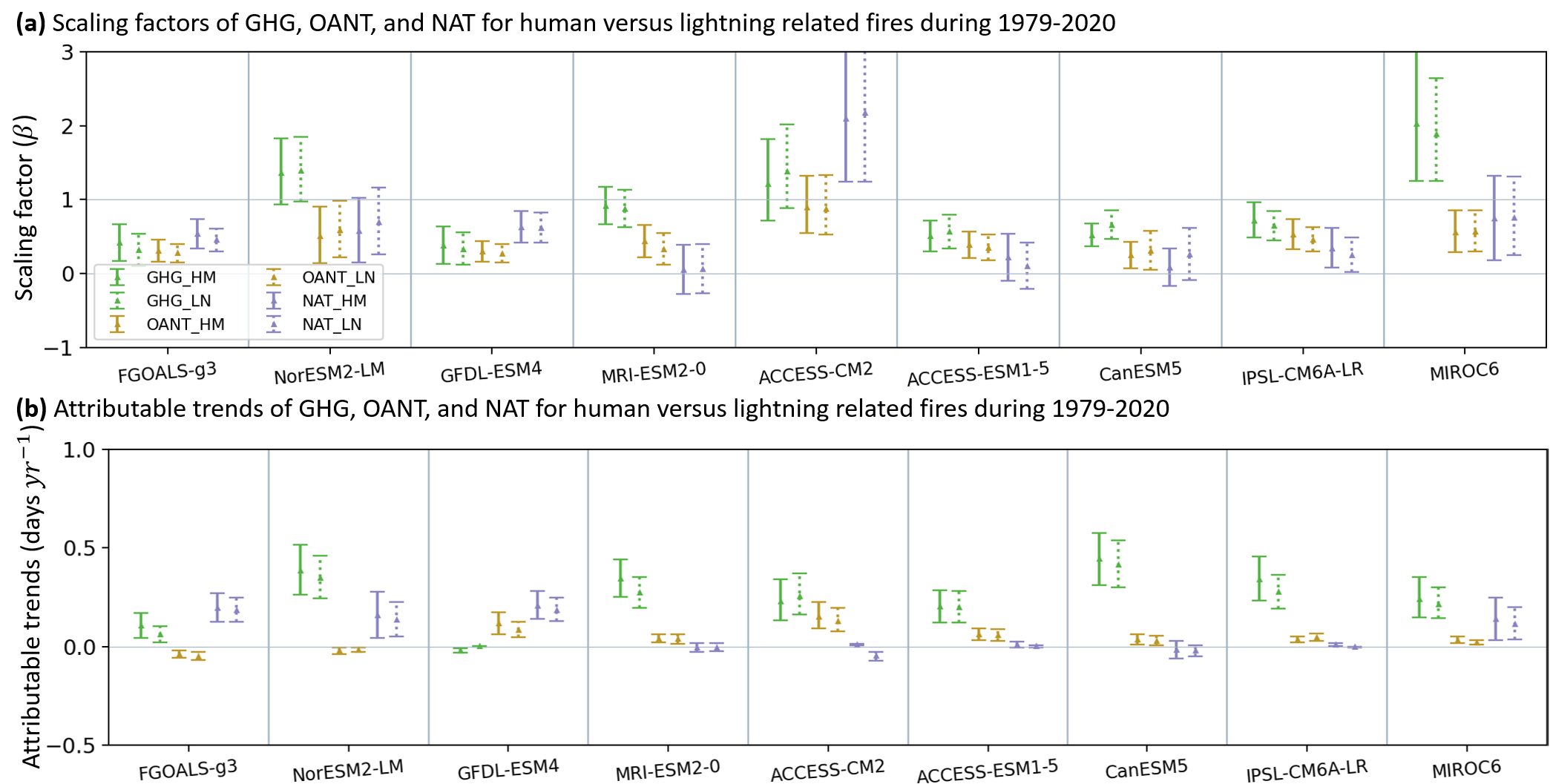
**

**Fig. S4**| Scaling factors **(a)** and attributable trends **(b)** derived from regression of observed anomalies in the frequency of flammable days onto three-signal forcings (GHG VS. OANT VS. NAT) for each Earth System Model. Error bars and triangles show 5%-95% confidence intervals and best estimates of scaling factors; confidence intervals above zero indicate a detectable (*p*<0.05) forced response, while confidence intervals containing zero indicate an undetectable forced response; the solid horizonal blue (red) line in (b) represents the observed trend for human (lightning) related frequency of flammable days. ‘LN’ and ‘HM’ represent scaling factors or attributable trends for lightning- and human-ignited large fires, respectively.


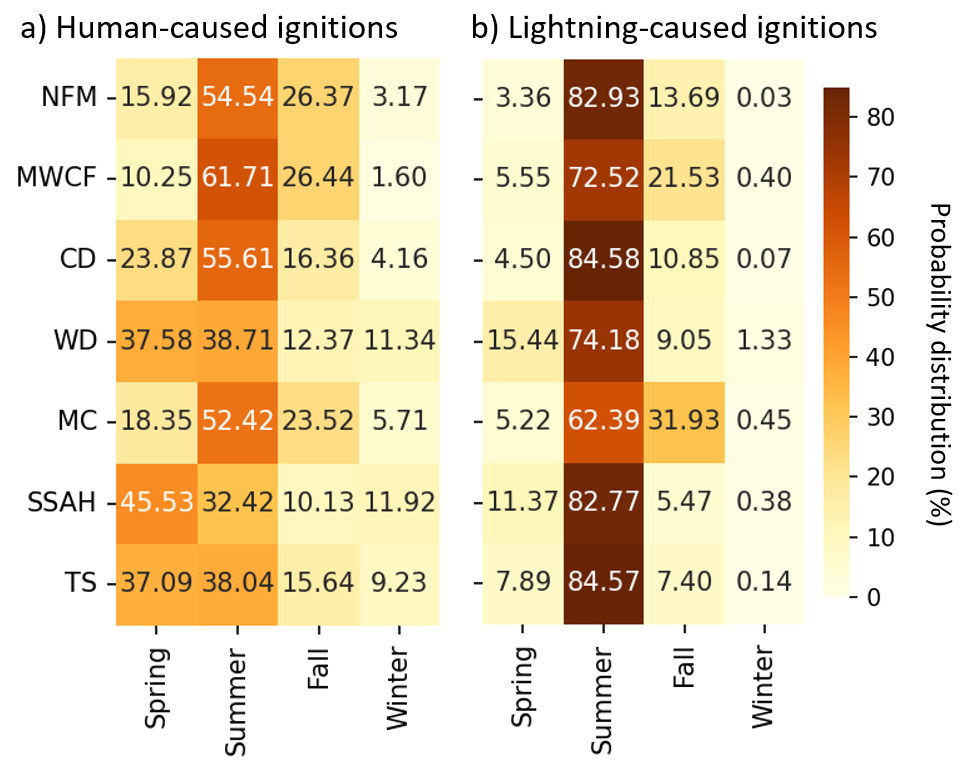


**Fig. S5**| Distribution of fire ignitions caused by humans **(a)** and lightning **(b)** over four seasons across different ecoregions in the western US.


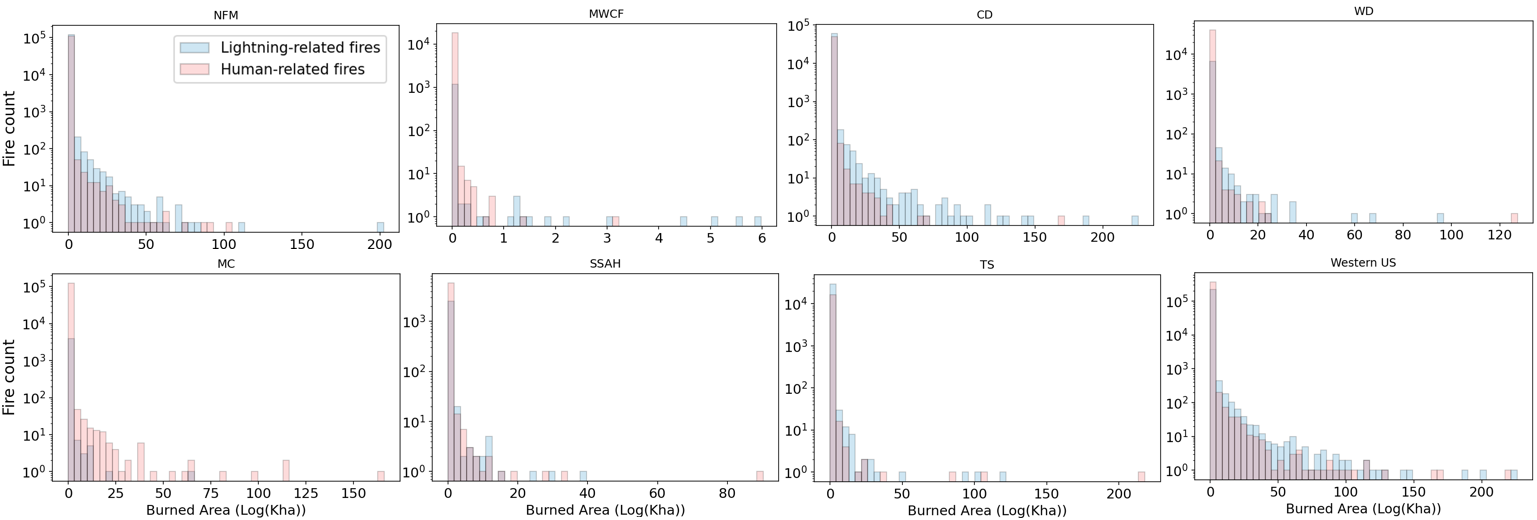


**Fig. S6**| Distribution of human- and lightning-ignited fires across various fire-size bins in each ecoregion and the western US. The burned area is log-transformed due to the wide range (e.g., fire size can differ by several orders of magnitude) and skew distribution (i.e., most fires have a relatively small size) of fire sizes.


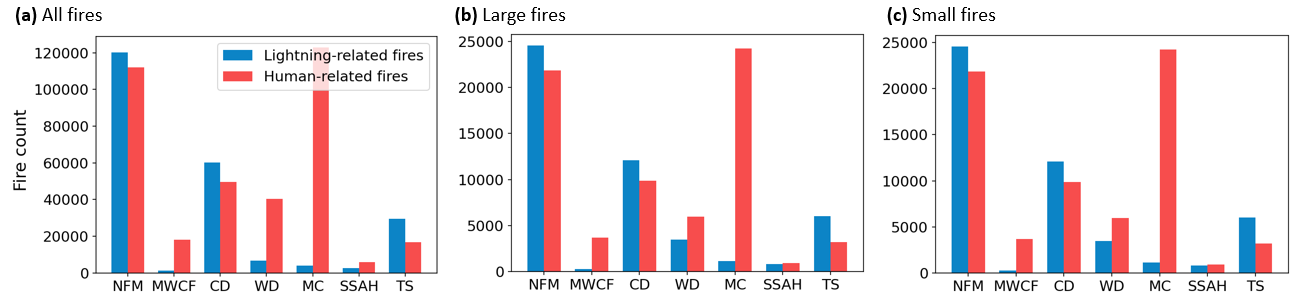


**Fig. S7**| Total number of human- and lightning-ignited fires across ecoregions in the western US **(a)**. Same to a) but for large fires **(b)**. Same to a) but for small fires **(c)**.

**References**

Balch, J. K., Abatzoglou, J. T., Joseph, M. B., Koontz, M. J., Mahood, A. L., McGlinchy, J., Cattau, M. E., and Williams, A. P.: Warming weakens the night-time barrier to global fire, Nature, 602, 442-448, 2022.

Bi, D., Dix, M., Marsland, S., O’Farrell, S., Sullivan, A., Bodman, R., Law, R., Harman, I., Srbinovsky, J., and Rashid, H. A.: Configuration and spin-up of ACCESS-CM2, the new generation Australian Community Climate and Earth System Simulator Coupled Model, Journal of Southern Hemisphere Earth Systems Science, 70, 225-251, 2020.

Boucher, O., Servonnat, J., Albright, A. L., Aumont, O., Balkanski, Y., Bastrikov, V., Bekki, S., Bonnet, R., Bony, S., and Bopp, L.: Presentation and evaluation of the IPSL‐CM6A‐LR climate model, Journal of Advances in Modeling Earth Systems, 12, e2019MS002010, 2020.

Bradshaw, L. S.: The 1978 national fire-danger rating system: technical documentation, US Department of Agriculture, Forest Service, Intermountain Forest and Range …1984.

Cherchi, A., Fogli, P. G., Lovato, T., Peano, D., Iovino, D., Gualdi, S., Masina, S., Scoccimarro, E., Materia, S., and Bellucci, A.: Global mean climate and main patterns of variability in the CMCC‐CM2 coupled model, Journal of Advances in Modeling Earth Systems, 11, 185-209, 2019.

Danabasoglu, G., Lamarque, J. F., Bacmeister, J., Bailey, D., DuVivier, A., Edwards, J., Emmons, L., Fasullo, J., Garcia, R., and Gettelman, A.: The community earth system model version 2 (CESM2), Journal of Advances in Modeling Earth Systems, 12, 2020.

Döscher, R., Acosta, M., Alessandri, A., Anthoni, P., Arneth, A., Arsouze, T., Bergmann, T., Bernadello, R., Bousetta, S., and Caron, L.-P.: The EC-earth3 Earth system model for the climate model intercomparison project 6, Geoscientific Model Development Discussions, 1-90, 2021.

Ellis, T. M., Bowman, D. M., Jain, P., Flannigan, M. D., and Williamson, G. J.: Global increase in wildfire risk due to climate‐driven declines in fuel moisture, Global change biology, 28, 1544-1559, 2022.

Flannigan, M., Wotton, B., Marshall, G., De Groot, W., Johnston, J., Jurko, N., and Cantin, A.: Fuel moisture sensitivity to temperature and precipitation: climate change implications, Climatic Change, 134, 59-71, 2016.

Gray, M. E., Zachmann, L. J., and Dickson, B. G.: A weekly, continually updated dataset of the probability of large wildfires across western US forests and woodlands, Earth System Science Data, 10, 1715-1727, 2018.

Held, I., Guo, H., Adcroft, A., Dunne, J., Horowitz, L., Krasting, J., Shevliakova, E., Winton, M., Zhao, M., and Bushuk, M.: Structure and performance of GFDL's CM4. 0 climate model, Journal of Advances in Modeling Earth Systems, 11, 3691-3727, 2019.

Jolly, W. M., Freeborn, P. H., Page, W. G., and Butler, B. W.: Severe fire danger index: A forecastable metric to inform firefighter and community wildfire risk management, Fire, 2, 47, 2019.

Jolly, W. M., Cochrane, M. A., Freeborn, P. H., Holden, Z. A., Brown, T. J., Williamson, G. J., and Bowman, D. M.: Climate-induced variations in global wildfire danger from 1979 to 2013, Nature communications, 6, 1-11, 2015.

Kelley, D. I., Bistinas, I., Whitley, R., Burton, C., Marthews, T. R., and Dong, N.: How contemporary bioclimatic and human controls change global fire regimes, Nature Climate Change, 9, 690-696, 2019.

Li, L., Yu, Y., Tang, Y., Lin, P., Xie, J., Song, M., Dong, L., Zhou, T., Liu, L., and Wang, L.: The flexible global ocean‐atmosphere‐land system model grid‐point version 3 (fgoals‐g3): description and evaluation, Journal of Advances in Modeling Earth Systems, 12, e2019MS002012, 2020.

Lovato, T., Peano, D., Butenschön, M., Materia, S., Iovino, D., Scoccimarro, E., Fogli, P., Cherchi, A., Bellucci, A., and Gualdi, S.: CMIP6 simulations with the CMCC Earth system model (CMCC‐ESM2), Journal of Advances in Modeling Earth Systems, 14, e2021MS002814, 2022.

Mauritsen, T., Bader, J., Becker, T., Behrens, J., Bittner, M., Brokopf, R., Brovkin, V., Claussen, M., Crueger, T., and Esch, M.: Developments in the MPI‐M Earth System Model version 1.2 (MPI‐ESM1. 2) and its response to increasing CO2, Journal of Advances in Modeling Earth Systems, 11, 998-1038, 2019.

Nolan, R. H., Boer, M. M., Resco de Dios, V., Caccamo, G., and Bradstock, R. A.: Large‐scale, dynamic transformations in fuel moisture drive wildfire activity across southeastern Australia, Geophysical Research Letters, 43, 4229-4238, 2016.

Seland, Ø., Bentsen, M., Olivié, D., Toniazzo, T., Gjermundsen, A., Graff, L. S., Debernard, J. B., Gupta, A. K., He, Y.-C., and Kirkevåg, A.: Overview of the Norwegian Earth System Model (NorESM2) and key climate response of CMIP6 DECK, historical, and scenario simulations, Geoscientific Model Development, 13, 6165-6200, 2020.

Swart, N. C., Cole, J. N., Kharin, V. V., Lazare, M., Scinocca, J. F., Gillett, N. P., Anstey, J., Arora, V., Christian, J. R., and Hanna, S.: The Canadian earth system model version 5 (CanESM5. 0.3), Geoscientific Model Development, 12, 4823-4873, 2019.

Tatebe, H., Ogura, T., Nitta, T., Komuro, Y., Ogochi, K., Takemura, T., Sudo, K., Sekiguchi, M., Abe, M., and Saito, F.: Description and basic evaluation of simulated mean state, internal variability, and climate sensitivity in MIROC6, Geoscientific Model Development, 12, 2727-2765, 2019.

Volodin, E., Mortikov, E., Gritsun, A., Lykossov, V., Galin, V., Diansky, N., Gusev, A., Kostrykin, S., Iakovlev, N., and Shestakova, A.: INM INM-CM5-0 model output prepared for CMIP6 CMIP historical, Version 20200601, 2019.

White, M. A., Thornton, P. E., and Running, S. W.: A continental phenology model for monitoring vegetation responses to interannual climatic variability, Global biogeochemical cycles, 11, 217-234, 1997.

Yukimoto, S., Kawai, H., Koshiro, T., Oshima, N., Yoshida, K., Urakawa, S., Tsujino, H., Deushi, M., Tanaka, T., and Hosaka, M.: The Meteorological Research Institute Earth System Model version 2.0, MRI-ESM2. 0: Description and basic evaluation of the physical component, Journal of the Meteorological Society of Japan. Ser. II, 2019.

Ziehn, T., Chamberlain, M. A., Law, R. M., Lenton, A., Bodman, R. W., Dix, M., Stevens, L., Wang, Y.-P., and Srbinovsky, J.: The Australian Earth System Model: ACCESS-ESM1. 5, Journal of Southern Hemisphere Earth Systems Science, 70, 193-214, 2020.
